# Combination of high‐throughput microfluidics and FACS technologies to leverage the numbers game in natural product discovery

**DOI:** 10.1111/1751-7915.13872

**Published:** 2021-06-24

**Authors:** Markus Oberpaul, Stephan Brinkmann, Michael Marner, Sanja Mihajlovic, Benedikt Leis, Maria A. Patras, Christoph Hartwig, Andreas Vilcinskas, Peter E. Hammann, Till F. Schäberle, Marius Spohn, Jens Glaeser

**Affiliations:** ^1^ Fraunhofer Institute for Molecular Biology and Applied Ecology (IME), Branch for Bioresources Giessen 35392 Germany; ^2^ Institute for Insect Biotechnology Justus‐Liebig‐University‐Giessen Giessen 35392 Germany; ^3^ Evotec International GmbH Göttingen 37079 Germany; ^4^ German Center for Infection Research (DZIF), Partner Site Giessen‐Marburg‐Langen Giessen 35392 Germany

## Abstract

High‐throughput platforms facilitating screening campaigns of environmental samples are needed to discover new products of natural origin counteracting the spreading of antimicrobial resistances constantly threatening human and agricultural health. We applied a combination of droplet microfluidics and fluorescence‐activated cell sorting (FACS)‐based technologies to access and assess a microbial environmental sample. The cultivation performance of our microfluidics workflow was evaluated in respect to the utilized cultivation media by Illumina amplicon sequencing of a pool of millions of droplets, respectively. This enabled the rational selection of a growth medium supporting the isolation of microbial diversity from soil (five phyla affiliated to 57 genera) including a member of the acidobacterial subgroup 1 (genus *Edaphobacter*). In a second phase, the entire diversity covered by 1071 cultures was used for an arrayed bioprospecting campaign, resulting in > 6000 extracts tested against human pathogens and agricultural pests. After redundancy curation by using a combinatorial chemical and genomic fingerprinting approach, we assigned the causative agents present in the extracts. Utilizing UHPLC‐QTOF‐MS/MS‐guided fractionation and microplate‐based screening assays in combination with molecular networking the production of bioactive ionophorous macrotetrolides, phospholipids, the cyclic lipopetides massetolides E, F, H and serratamolide A and many derivatives thereof was shown.

## Graphical abstract

This study details our approach in establishing a high‐throughput droplet microfluidics cultivation platform combined with fluorescence‐activated cell sorting (FACS), which enabled the efficient retrieval, cultivation and arraying of axenic cultures from a complex environmental sample. Our cultivation platform utilizes agarose‐solidified microspheres showing long‐term integrity, while possessing flexibility to adapt the experimental set‐ups towards manifold metabolic needs of microorganisms. Modulation of media compositions and especially pH altered the composition of the cultured diversity and resulted in the isolation of strains from underrepresented phyla such as Acidobacteria as well as scarcely‐cultured Proteobacteria (*Ancylobacter*, *Buttiauxella*, *Inquilinus*, *Kaistia*, *Labrys*, *Luteibacter*, *Polaromonas*, *Reyranella* and *Variovorax*). Illumina amplicon sequencing was used to rate the diversity within the bulk of droplets and furthermore to guide the sample selection for a miniaturized and largely automated natural product discovery campaign including > 6000 extracts retrieved from 1071 cultures.

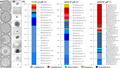

**Originality Significance Statement**

Environmental habitats and their microbiota represent the most valuable sources for the identification of natural products. The proven biosynthetic potential of talented natural product producers and yet‐uncultured microorganisms constitute a large chemical space, waiting to be further explored. Thus, it is mandatory to constantly expand the accessible taxonomic diversity. Miniaturized, micron‐scaled cultivation platforms offer solutions to massively parallelize and efficiently promote cultivation campaigns. Advanced microfluidics technologies themselves depict an environmental mimicry and allow the microorganisms to adapt the conditions towards their specific metabolic needs. Applied as first step of a natural product discovery platform, we obtained axenic cultures from a soil habitat and integrated the cultivated diversity into a bioactivity‐guided bioprospecting programme. The assessment of unique strains and their organic extracts showing bioactivity streamlined sample prioritization and identification of the causative natural products. The herein reported platform is optimized to access and characterize microbial diversity from environmental samples and their exhibited chemical space. Its robustness and flexibility allow its translation to a diversity of bioresources and research disciplines, contributing to advancing efficient exploitation of the microbial dark matter.

## Introduction

Continuously increasing levels of drug resistance and the consequent loss of existing drug and control agents for the treatment of infections poses an enormous threat to human health care systems and plant disease management strategies (Lewis, 2013; Lakemeyer *et* *al*., 2018). This crisis has to be counteracted by continued innovation in discovery campaigns to increase the probability of finding new anti‐infective lead structures (Schäberle and Hack, 2014; Tacconelli *et* *al*., 2018; Theuretzbacher *et* *al*., 2020).

A rich source for new chemical entities are microbial‐derived natural products (NPs), which always has been a major inspiration for development of drugs and control agents (Newman and Cragg, 2020). NP evolution brought forth a vast diversity of unique molecules that are optimized for interactions with their respective molecular targets; the latter a plethora of biological macromolecules themselves (Firn and Jones, 2003; Bon and Waldmann, 2010; Hong, 2011). Fortunately, the predicted potential for chemical variety encoded within the microbial diversity is yet only scarcely exploited. This is mainly due to the fact that major NP discovery efforts of past decades had been focusing on rather easily cultivable microorganisms and consequently on a limited phylogenetic diversity (Monciardini *et* *al*., 2014). Along with the likely also not yet‐exhausted potential of classical NP producers, for example *Streptomyces* spp. (Adamek *et* *al*., 2019; Belknap *et* *al*., 2020), additional phylogenetic branches of the bacterial kingdom represent a promising source for discovery of novelty (Gross and Loper, 2009; Panthee *et* *al*., 2016; Tracanna *et* *al*., 2017). Thus, driven by the dogma that phylogenetic and genomic divergence translates directly into chemical diversity (Medema *et* *al*., 2014; Monciardini *et* *al*., 2014), greater effort is demanded to bring a broader diversity of genera and families in culture (Hoffmann *et* *al*., 2018; Nicault *et* *al*., 2020) while directly exploiting their metabolic capabilities. The still valid functionality of the methodology to valorize bacterial derived NPs was recently shown by the development and market introduction of fenpicoxamid, derived from the antifungal compound UK‐2A produced by *Streptomyces* sp. 517‐02. This NP provides a new target site for the control of *Zymoseptoria* *tritici* (SEPTTR), the causative agent of Septoria tritici Blotch (STB) (Butler and Paterson, 2020) by inhibiting the mitochondrial complex III at a target site distinguished from the one attacked by the strobilurin class (Owen *et* *al*., 2017). The trend towards the valorization and application of naturally derived fungicides continues to rise, predicted to obtain further attention in the next decades (Umetsu and Shirai, 2020). In antibacterial research, success in discovery of novel NPs with unique modes of action was recently mainly achieved with the isolation of novel anti‐infective lead structures from rare Proteobacteria, particularly darobactin (from *Photorhabdus* sp.), a heptapeptide antibiotic targeting the gram‐negative outer membrane protein *BamA* (Imai *et* *al*., 2019) and teixobactin (from *Eleftheria* sp.), which was made accessible by a novel cultivation technique (Ling *et* *al*., 2015).

The basis for all such findings is the fundamental accessibility to a broad, thus diverse phylogenetic space of the bacterial kingdom. The challenging task to bring an additional layer of today’s microbial dark matter into culture is mainly a numbers game that has to be approached by the development and implementation of new and efficient methods (Lok, 2015). Microbial cultivation strategies, with their common theme in mimicking environmental conditions in laboratory settings (Kaeberlein, 2002), require massive miniaturization and brute force cultivation techniques while still considering the microorganisms’ metabolic needs (Keller and Zengler, 2004). Advanced miniaturization approaches are microfluidics‐based strategies, posing a rapidly emerging technology for cultivation of microbial diversity that were also already utilized in the field of NP discovery (Zinchenko *et* *al*., 2014; Mahler *et* *al*., 2015; Terekhov *et* *al*., 2017). Droplet microfluidics is based on converging aqueous and oil phases in a laminar flow with the addition of surfactants as stabilizing agents. Pressurized in microchannels with a diameter ranging from 30 to 500 µm, this ultimately results in aqueous droplets in nl to pl scale (Leman *et* *al*., 2015). The production of millions of droplets per hour was improved over years, and simplified systems are nowadays on the market (Nge *et* *al*., 2013; Volpatti and Yetisen, 2014). Their combination with further technologies, such as fluorescence‐activated cell sorting (FACS), enables the direct identification of desired events and their arrayed sorting (Zinchenko *et* *al*., 2014). An advantageous value of these technologies is the downscaling and compartmentalization which allows single cells to be physically separated into distinct vessels while maintaining the overall microbial complexity (Zengler *et* *al*., 2002; Theberge *et* *al*., 2010). This separation is mandatory to avoid growth competition between different species while also allowing single cells to utilize and shape their microenvironment according to their specific needs and at their own pace (Keller and Zengler, 2004; Boitard *et* *al*., 2015). The pico‐litre scaled droplets display an environmental mimicry themselves, since the extremely small scale enables even single cells to adapt their environment by, for example accumulating self‐mediating growth factors eventually breaking microbial dormancy (Boedicker *et* *al*., 2009; Ishii *et* *al*., 2010; Stewart, 2012). In principal, high‐throughput microfluidic‐based platforms increase the microbial cultures in amount but also diversity and consequently the probability to cultivate and identify also underexplored microorganisms (Akselband *et* *al*., 2006; Baret *et* *al*., 2009).

In this study, we succeeded in isolating an extended taxonomic diversity and accessing microorganisms considered as under‐ or even unexploited for NP discovery. We implemented a biphasic workflow consisting of (i) an efficient cultivation of microbial diversity using microfluidics and FACS‐based technologies, followed by (ii) a miniaturized bioactivity‐guided NP discovery process on the obtained isolates focused on the two devastating pathogens, that is *Mycobacterium* *tuberculosis* (MTB) and SEPTTR. Our here described cultivation platform is based on long‐term stable agarose‐solidified microdroplets (~ 40 µm in size, volume of ~ 33 pl). Droplets were generated up to rates of ~ 1.3 kHz; thereby, exceeding the dimensions for an application to be affiliated as ultra‐high throughput (Payne *et* *al*., 2020). At a cell distribution of λ0.1, this set up allowed the parallel encapsulation of ~ 500 000 cells h^−1^ with a statistical probability below 0.5% to obtain co‐cultures. In total, we brought 1071 microorganisms into culture, from which ~ 74% could be identified by 16*S* rRNA gene sequencing. The isolated bacteria belong to five different phyla affiliated to 57 different genera. Besides classical NP producing taxa a representative of fastidious Acidobacteria and certain proteobacterial genera which are underrepresented in public strain libraries (e.g. *Luteibacter* and *Variovorax*) were brought into culture. The entire obtained microbial diversity was eventually integrated into a bioactivity‐guided NP discovery process. After redundancy curation of initially active extracts, the organic extracts of *Erwinia*, *Pseudomonas* and *Streptomyces* which most strongly inhibited the growth of MTB and/or SEPTTR were followed up by bioassay‐ and UHPLC‐QTOF‐MS/MS‐guided fractionation. Complemented by extensive metabolomic analysis via molecular networking, this led to the identification of serratamolides, massetolides, phospholipids and macrotetrolides as the bioactivity causing agents.

## Results and discussion

### Cultivation of microbial diversity using microfluidics and FACS

In this study (platform scheme see Fig. S2), we aimed to bring microbial diversity into culture to expand our strain collection (Fox, 2014) and to exploit the bacterial proportion in a bioactivity‐guided NP discovery programme. Known for their highly diverse bacterial communities (Delgado‐Baquerizo *et* *al*., 2016), we selected a combined soil sample as starting material for our study.

**Fig. 1:**
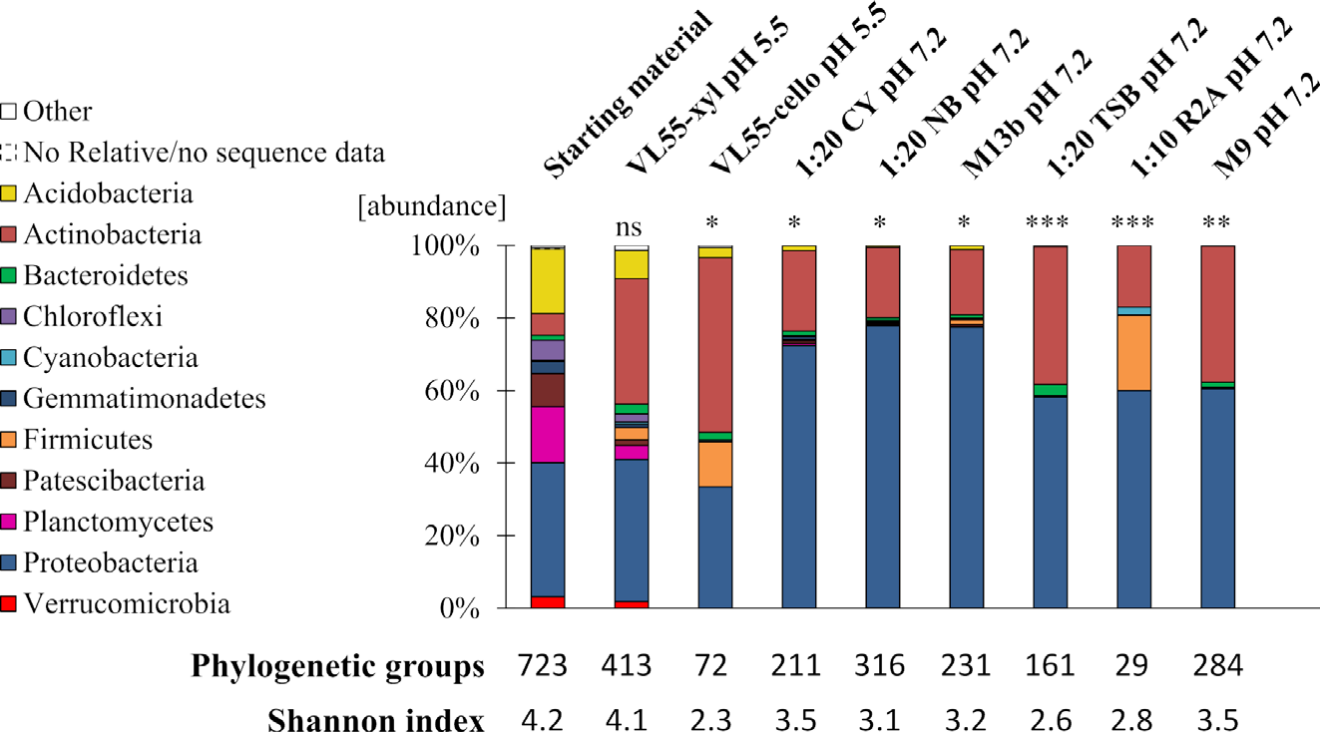
Microbial diversity grown in agarose‐solidified droplets by Illumina amplicon sequencing proves VL55‐xyl as best cultivation media to obtain highest diversity. Isolated environmental cells (starting material) were encapsulated in droplets (λ0.1) and incubated at 28°C for seven days before total DNA was directly extracted from the droplet populations and sequenced with Illumina 300‐bp paired‐end 16*S* V3‐V4 amplicon next‐generation sequencing, respectively. VL55‐xyl outperformed with the highest number of phylogenetic groups (491) and Shannon index on genus level of 4.1. A significant difference between media sample medians was observed on phylum level (*P* > 0.005). Dunn’s post hoc test was performed on all samples on phylum level compared to the starting material (***P < 0.001; **P < 0.01; *P < 0.1; ns: not significant).

Upfront the main droplet cultivation and screening campaign, we examined the general cultivation success using our microfluidics platform and explicitly the specific impact of the chosen growth media on the bacterial community. Specifically adapted towards soil and previously shown to be suitable to transfer a high diversity of soil microorganisms into culture, we selected ISEM (Nguyen *et* *al*., 2018) as benchmark medium for this study. Considering the importance of using buffered media as well as mimicking neutral and acidic soils for the cultivation success (Overmann *et* *al*., 2017), we buffered the media to pH 7.2 and pH 5.5 (Tovar *et* *al*., 2020). In order to identify an additional media suitable to access a broad microbial diversity of the bioresource used in this study, we encapsulated the retrieved microorganisms (λ0.1) in eight different media (VL55‐xyl, VL55‐cello, 1:20 CY, 1:20 NB, M13b, 1:20 TSB, 1:10 R2A and M9). Then, we comparatively determined the cultivation success by Illumina amplicon sequencing of the sorted droplet populations among themselves and towards the starting material. The sequencing was conducted on DNA directly isolated from the eight different droplet populations. Illumina amplicon sequencing of isolated DNA yielded in total 1 947 118 classified sequences, of which 0.04% could not be classified and were defined as *No* *Relative*. The data revealed that cultivation in VL55‐xyl resulted in the overall highest diversity on genus level resolution (Shannon index [SI]: 4.1), followed by M9 (SI: 3.5) and 1:20 CY (SI: 3.5) (Fig. 1). Compared to the starting material, the microbial diversity covered by VL55‐xyl was not significantly reduced (*P* > 0.5). In contrast, the exchange of the sole C‐source xylan by cellobiose led to a significant reduction in diversity (*P* < 0.01) and a lower SI (SI: 2.3). While showing an overall lower diversity, the media (1:20 CY, 1:20 NB, M13b, 1:20 TSB and 1:10 R2A), which contained complex components such as yeast or peptone showed an increased proportion of Proteobacteria (up to 77.7%) in comparison with the VL55‐based media (up to 39.2%) and the starting material (36.7%). A similar diversity and an increased proportion of Proteobacteria were observed by using M9. While 1:10 R2A appears to be suitable to enrich Firmicutes (20.8% vs 0.06% in starting material), all other media buffered at pH 7.2 do not show a remarkable difference within their cultivated microbial community (*P* > 0.7; Fig. 1).

Overall, VL55‐xyl with its complex C‐source and its pH favourable for acidophilic and acid‐tolerant bacteria was shown to be the most suitable medium for our soil material and selected it to be included in all subsequent experiments. Considering the observed effects of the Illumina analysis, particularly the impact of the provided C‐source and the altered pH profile, we supplemented ISEM with a variation of six complex C‐sources including xylan (mISEM^1^) and buffered it to pH 5.5 and pH 7.2.

**Fig. 2:**
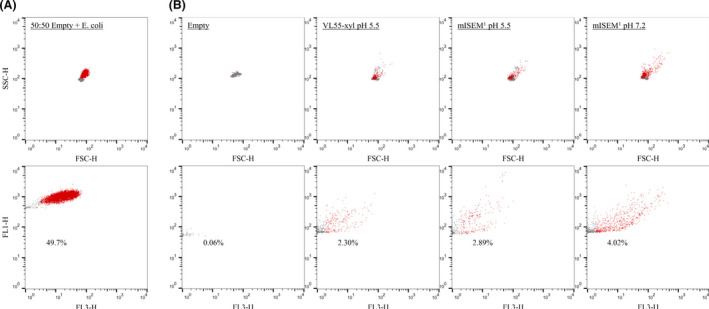
Sorting of microcolonies grown in agarose‐solidified droplets stained using DiOC_2_(3). A. Differentiation of a 50:50 mixture of empty droplets and those containing grown microcolonies of *E*. *coli* ATCC 25922 (λ10). 15 000 events were analysed by FACS considering granularity (SSC‐H) to size (FSC‐H) and green (FL1‐H) to red fluorescence (FL3‐H) after staining. B. Sorting of droplets containing grown microcolonies of environmental soil microorganisms (λ0.1) in VL55‐xyl pH 5.5, mISEM^1^ pH 5.5 and mISEM^1^ pH 7.2 (10 000 events displayed). The background noise was 0.06% (empty = control without cells encapsulated). After seven days of incubation at 28°C, grown microcolonies as determined by membrane potential dye were sorted and recovered. Red dots = droplets containing fluorescently labelled cells; grey dots = empty droplets.

In this study, we applied a micron‐scaled cultivation platform to receive axenic cultures from a complex bioresource, adapted for the purpose to also cultivate slow‐growing microorganisms. This necessitates the analysis of droplets in high‐throughput fashioned manner and the reliable discrimination between droplets harbouring grown microorganisms with manifold morphologies and the empty droplet background. Therefore, we used a FACS approach utilizing the carbocyanine dye DiOC_2_(3) to stain microorganisms with a membrane potential grown inside the agarose‐solidified droplets. DiOC_2_(3) is a potentiometric probe that exhibited green fluorescence in all bacterial cells; however, the fluorescence shifts towards red emission as the dye molecules self‐associate at the higher cytosolic concentrations caused by larger membrane potentials (Shapiro, 2000; Biener *et* *al*., 2017). To show the feasibility of using DiOC_2_(3) for this process step, a mixed droplet population containing 50% droplets harbouring grown microcolonies of *E*. *coli* (λ10) incubated for 4 h at 37°C and 50% empty droplets was stained and subsequently measured using FACS while analysing the ratio of green (FL1‐H) and red (FL3‐H) fluorescence. The FL1‐H to FL3‐H scatter exposed 49.7% of all analysed events in the defined gate, congruent with the adjusted 50.0% of the droplet population containing grown *E*. *coli* microcolonies (Fig. 2A). This verified the applicability of DiOC_2_(3) as a fluorescent dye for staining microorganisms grown in agarose‐solidified droplets. Based on these distinguishable populations, the FACS settings (e.g. sorting gates for events of interest) were defined and later on applied for the cultivation campaign.

**Fig. 3:**
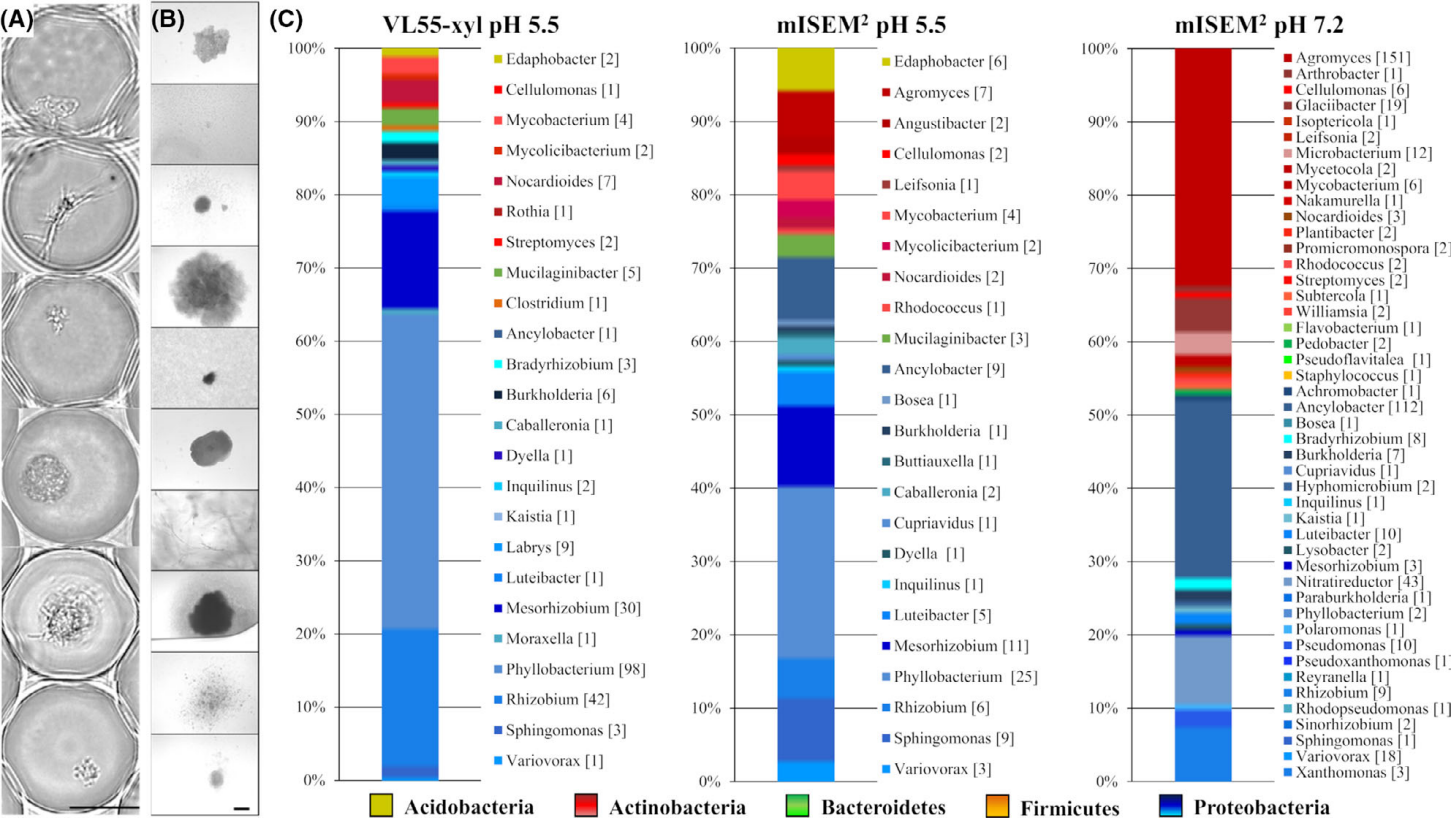
Successful cultivation of microbial diversity after incubation of solidified‐agarose droplets verified by 16*S* rRNA gene sequencing (1492R primer) and microscopy. A. Examples of grown microcolonies in agarose‐solidified droplets were microscopically examined. Scale bar: 20 µm. B. Inverse microscopy of exemplary 384‐well MTPs containing arrayed droplets and liquid medium. Different morphologies from microcolonies, which grew out of droplets are indicated, representing a section of the final retrieved and conserved bacterial diversity. Scale bar: 40 µm. C. In total, 1071 MOs affiliated to 5 phyla were brought into culture, whereas ~ 74% were initially identified by 16*S* rRNA gene sequencing (partial sequence using primer 1492R, mISEM^2^ pH 7.2: 462, mISEM^2^ pH5.5: 106, VL55‐xyl pH5.5: 225). Numbers in square brackets indicate the amount of 16*S* rRNA gene sequencing affiliations. For further information and data used for the figure caption, see Table S1.

The cell suspension from the used soil sample retrieved using nycodenz gradient centrifugation had an estimated living cell count of ~ 4.2 × 10^6^ cells ml^−1^ determined via FACS [considering live/dead ratio (~ 70:30; Fig. S3)]. This was taken into consideration, while cells were encapsulated in different media (λ0.1 each) and subsequently incubated at 28°C, respectively. The microbial growth within droplets was frequently monitored over time by microscopy, exemplarily showing the processing of microorganisms with various morphologies (Fig. 3A). After seven days of incubation, the samples were sorted by using DiOC_2_(3) staining. We could affiliate 2.30% (VL55‐xyl pH 5.5), 2.89% (mISEM^2^ pH 5.5) and 4.02% (mISEM^2^ pH 7.2) of all evaluated events to droplets containing grown microcolonies (Fig. 2B). These events were sorted and distributed into 384‐well MTPs, and microbial growth was again monitored by turbidimetry and microscopy, respectively (Fig. 3B). After automatic transfer of grown cultures into 96‐deep well MTPs and further incubation for up to 14 days, this up‐scaling procedure provided 1071 cultures in total. Sample aliquots of each culture were taken to generate cryo‐conserved cultures and cell lysates for taxonomic identification by 16*S* rRNA gene sequencing (partial sequencing using primer 1492R). This revealed the processing of 57 different genera affiliated to five different phyla (Fig. 3C and Table S1). Comparing the cultivation, success of mISEM^2^ at pH 5.5 and pH 7.2 clearly shows the worth of modulating this parameter to increase the cultivated diversity. Together, 55 genera were isolated using mISEM^2^, while only 15 of them could be found under both pH conditions. Medium mISEM^2^ pH 7.2 led to the successful cultivation and affiliation of 46 genera (462 strains, SI: 2.4) while 31 genera were unique to this condition. In contrast, at pH 5.5 a total of 24 genera (108 strains, SI: 2.7) and 9 unique ones were identified. While use of acidified media led to an overall decreased amount of genera, this condition showed a higher diversity and expanded the cultivation success towards a fifth phyla (Acidobacteria) not present at the neutral pH set‐up. Within both media comprising pH 5.5, we found eight representatives of the Acidobacteria, all belonging to the genus *Edaphobacter*. A representative thereof was recovered on plate and incorporated into our strain collection. As judged on nearly full‐length 16*S* rRNA sequence comparison, this particular strain, namely FHG110552, is phylogenetically affiliated to the subgroup 1 of Acidobacteria and most closely related to *Edaphobacter* *modestus* Jbg‐1T (~ 98.8% similarity) isolated from alpine and forest soils (Koch *et* *al*., 2008; Fig. S4). The phylum Acidobacteria is postulated to be a promising bioresource for the field of NP discovery based on its predicted biosynthetic potential encoded in their genomes (Kielak *et* *al*., 2016; Crits‐Christoph *et* *al*., 2018). Though, the description of Acidobacteria NPs is yet limited to hopanoids (Damsté *et* *al*., 2017).

On phylum level, a major difference between the neutral and acidified media is the ratio between Actinobacteria and Proteobacteria, which is at pH 7.2 shifted towards the Actinobacteria, while towards Proteobacteria at pH 5.5. The same is seen in VL55‐xyl covering in total 23 genera (223 strains, SI: 1.9) including *Edaphobacter* but also unique genera such as *Labrys* and *Rothia*, not covered by mISEM^2^. In total, 17 Actinobacteria genera were brought into culture using mISEM^2^ pH 7.2, whereas 8 genera in mISEM^2^ pH 5.5 and 6 genera in VL55‐xyl. This is particularly biased by the genus *Agromyces*, which was strongly enriched in mISEM² pH 7.2 (151 strains) while present in low abundance at pH 5.5 (7 strains).

Within the Proteobacteria, such an opposite pH‐dependent shift occurs within the class of Alphaproteobacteria. At pH 7.2, the genus *Ancylobacter* represents almost half of all Proteobacteria (112 strains) while being significantly less abundant at pH 5.5. In contrast, the genus *Phyllobacterium* represents > 30% of the Proteobacteria at acidic condition while < 1% at neutral pH. In total, the isolated Proteobacteria proportion comprises member of 33 different genera belonging to the classes of Alpha‐, Beta‐ and Gammabacteria and include also representatives of nine scarcely cultured genera according to our definition (namely *Ancylobacter*, *Buttiauxella*, *Inquilinus*, *Kaistia*, *Labrys*, *Luteibacter*, *Polaromonas*, *Reyranella* and *Variovorax*).

### Bioactivity‐guided bioprospecting process

To prospect the here cultured microbial diversity for NPs with desired bioactivity, we integrated all arrayed microbes in a downscaled bioactivity‐guided NP discovery process. Each culture was grown in its respective isolation medium and in addition in ISP2 and BSM for four and seven days. This process aimed to more sophistically provoke expression of the microbial secondary metabolite production potential. Using 96‐deep well Duetz systems and 1 ml culture volumes, 6426 organic extracts were generated and used for bioactivity screenings and metabolome analysis.

**Fig. 4:**
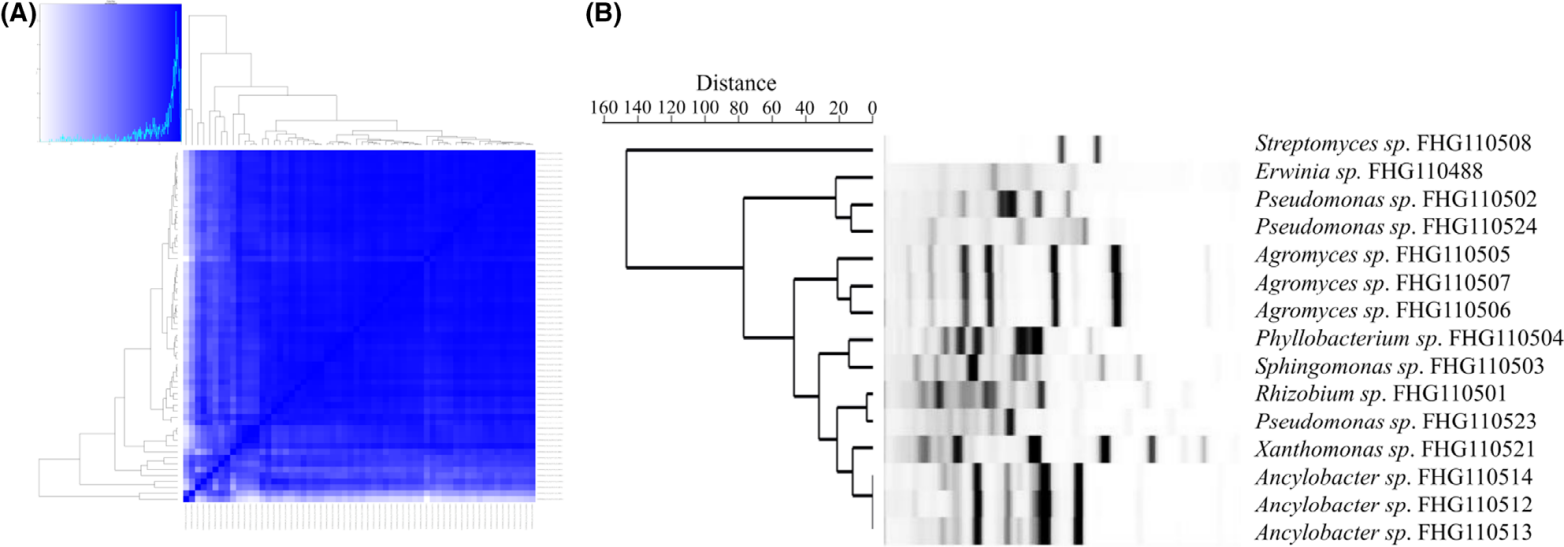
Metabolic grouping of extracts based on the cosine similarity of their associated UHPLC‐UHR‐MS chromatograms and the respective genetic fingerprinting by BOX‐PCR applied to reduce the redundancy of active strains. (A) Metabolic clustering of extracts of all bioactive strains was analysed via LC‐MS. The cosine similarities between samples were calculated. Cosine similarity values for strain‐condition pairs were extracted. Samples were sorted according to clustering results, and pairwise similarities were used to define metabolic groups. If the pairwise similarity between two subsequent clustered samples was at the threshold 0.9 or higher, they were assigned to one metabolic group. Figure caption can be found in Table S2. (B) Repetitive genome sequences of all strains initially found to be bioactive were targeted by BOX‐PCR to identify unique strains. [Correction added on 01 July 2021, after first online publication: Figure 4 legend has been amended for clarity in this version]

In first line, the extracts were screened for their antimycobacterial activity against *M*. *smegmatis*, the opportunistic microbial pathogen *S*. *aureus* and for their antifungal activity against *Z*. *tritici*, *C*. *albicans* and *A*. *flavus*. Extracts that showed growth inhibitory properties underwent a subsequent diversity assessment by cosine similarity comparison of features detected within each corresponding UHPLC‐UHR‐MS chromatogram. This quality control step was included to rule out extensive analysis of redundant samples, unavoidably appearing in every microbial isolation project. After screening the methanolic extracts, 64 cultures were assigned to bioactivity by either inhibiting the growth of *M*. *smegmatis* and/or the fungal indicator strains. The cosine similarity of their associated UHPLC‐UHR‐MS chromatograms were determined. After excluding samples with less than 50 features, the remaining 60 samples clustered into eight distinct groups applying the cosine similarity threshold 0.9 (Fig. 4A, Table S2). From the most dominant group, we recovered *Penicillium* sp. (FHG110518). With no focus on fungal metabolites in this project, we decided to discontinue the work on this abundant strain causing the major fractions of observed bioactivities (~ 70%). In order to now identify the bacterial strains in this dataset, we complemented the redundancy curation process by genotyping the cultures using BOX‐PCR (Fig. 4B). By superimposing the patterns of both grouping technologies, we identified and subsequently recovered strains of the genera *Pseudomonas* [FHG110502, FHG110523 and FHG110524 (each unique)], *Xanthomonas* (FHG110521), *Rhizobium* (FHG110501), *Sphingomonas* (FHG110503), *Phyllobacterium* (FHG110504), *Ancylobacter* [FHG110512, FHG110513, FHG110514 (equal)], *Agromyces* [FHG110505, FHG110506, FHG110507 (equal)], *Erwinia* (FHG110488) and *Streptomyces* (FHG110508).

In order to identify the causative NP beyond the observed crude extract activities, the cultivation of the unique strains was scaled‐up to 50 ml volumes. The resulting culture broths were extracted by methanol and reconstituted in DMSO for bioactivity screenings. The extracts from the three strains *Pseudomonas* sp. (FHG110502), *Erwinia* sp. (FHG110488) and *Streptomyces* sp. (FHG110508) exhibited the severest growth inhibitory potency and were selected for further analysis. Their crude extract complexity was strongly reduced by their separation into 159 fractions. After rescreening all fractions, the compounds present in the active fractions were identified by their exact masses, characteristic UV absorption spectra and fragmentation signature.

#### *Erwinia* sp. FHG110488

Based on the primary activity of the crude extract against *M*. *smegmatis* and *Z*. *tritici*, the corresponding extract was fractionated and rescreened. Compound **1** with a *m*/*z* of 515.3329 [M + H]^+^, corresponding to the molecular formula C_26_H_46_N_2_O_8,_ was dereplicated within an active fraction (Fig. S5). On the basis of the molecular formula and the observed MS/MS fragmentation pattern, the bioactive compound was dereplicated as the known serratamolide A (Wasserman *et* *al*., 1961; Dwivedi *et* *al*., 2008). Using MS/MS networking, six additional derivatives were identified (measured **2** *m*/*z* 541.3480 [M + H]^+^, C_28_H_48_N_2_O_8_, theoretical *m*/*z* 541.3483; **3** *m*/*z* 543.3635 [M + H]^+^, C_28_H_50_N_2_O_8_, theoretical *m*/*z* 543.3639; **4** *m*/*z* 533.3430 [M + H]^+^, C_26_H_48_N_2_O_9_, theoretical *m*/*z* 533.3432; **5** *m*/*z* 547.3588 [M + H]^+^, C_27_H_50_N_2_O_9_, theoretical *m*/*z* 547.3589; **6** *m*/*z* 575.3896 [M + H]^+^, C_29_H_54_N_2_O_9_, theoretical *m*/*z* 575.3902; **7** *m*/*z* 573.3745 [M + H]^+^, C_29_H_52_N_2_O_9_, theoretical *m*/*z* 573.3745 (Fig. S6). Serratamolide A is a biosurfactant with plant protecting properties (Thies *et* *al*., 2014), additionally alleviating uptake of substances due to its amphiphilic wetting effect, lowering surface and interfacial tensions (Mulligan, 2005). It is known to show antioomycetous activity with particular efficacy against *Pythium* *ultimum* and *Phytophthora* *parasitica* (Strobel *et* *al*., 2005), a further devastating pest with lack in effective control measures under investigation (Pöppel *et* *al*., 2014).

#### *Pseudomonas* sp. FHG110502

The antimycobacterial activity caused by *Pseudomonas* strain FHG110502 was associated to three cyclo‐lipo‐nonadepsipeptide **8–10** (**8** *m*/*z* 1112.6811 [M + H]^+^, C_53_H_93_N_9_O_16_, theoretical *m*/*z* 1112.6813; **9** *m*/*z* 1126.6966 [M + H]^+^, C_54_H_95_N_9_O_16_, theoretical *m*/*z* 1126.6969; and **10** *m*/*z* 1154.7288 [M + H]^+^, C_56_H_99_N_9_O_16_, theoretical *m*/*z* 1154.7282) perfectly matching the MS/MS fragmentation signature of massetolide E, F and H, respectively. Massetolide E and F differ in AA9 by incorporation of valine and leucine, respectively. Massetolide H differs in the fatty acid chain. The hydroxydecanoic acid residue of E and F is replaced by a hydroxylauric acid residue (Fig. S7). At position AA9, the known antimycobacterial massetolide family is known to be prone to natural variations, with indications in a general correlation between greater lipophilicity and increased potency (Gerard *et* *al*., 1997).

Furthermore, three phospholipids dereplicated as lyso‐palmitoyl‐phosphoethanolamine (**11**), palmitoleoyl‐palmitoyl‐phosphoetanolamine (**12**) and palmitoleoyl‐oleoyl‐phosphoethanolamine (**13**) (measured **11** *m*/*z* 454.2931 [M + H]^+^, C_21_H_44_N_1_O_7_P_1_, theoretical *m*/*z* 454.2928; **12** *m*/*z* 690.5073 [M + H]^+^, C_37_H_72_N_1_O_8_P_1_, theoretical *m*/*z* 690.5068; and **13** *m*/*z* 716.5237 [M + H]^+^, C_39_H_74_N_1_O_8_P_1_, theoretical *m*/*z* 716.5224), respectively, were assigned the other active fractions (Fig. S8). The bioactivity of phospholipids against serval indicator strains has been reported, for example bacilysocin as an antifungal active phospholipid isolated from *Bacillus* *subtilis* (Tamehiro *et* *al*., 2002).

#### *Streptomyces* sp. FHG110508

A classical NP producer organism that was isolated during our process is *Streptomyces* FHG110508. Its organic extracts showed pronounced activity against *S*. *aureus* and the fungal indicator strains. Dereplication of active fractions revealed the presence of the macrotetrolides nonactin, monactin, dinactin and macrotetrolide G (Fig. S9, **14–17**) (Phillies, 1975; Řezanka *et* *al*., 2010). Additionally, the presence of macrotetrolide D (**18**) was shown by molecular networking (Fig. S10). Ionophore antibiotics of the macrotetrolide family are beyond the most commonly observed bioactive metabolites from actinomycetes passing in various screening disciplines having antibacterial, antifungal, antiprotozoan, antiparasitic, insecticidal and acaricidal activity (Zizika, 1998).

#### Conclusive remarks

Our study represents a combination of applied microfluidics and FACS technologies for the cultivation of microorganisms in agarose‐solidified droplets. The combination of streamlined high‐throughput technologies facilitated the generation and analysis of thousands of droplets within seconds. This paved the way to a fast isolation and characterization of diverse axenic cultures, followed by downstream identification of bioactive natural products. There are no limits on a particular bioresource, organism types or cultivation conditions (media, nutrient limitation, temperature, incubation method, ±oxygen supply, etc.), since especially all parameters towards cultivation in solidified droplets are highly customizable. Microfluidic platforms are already today a core element to access and assess microbial diversity from environmental samples. Their efficient application on a diversity of bioresources will be of steadily increasing importance for various research disciplines, with particular (but not exclusive) regard to the field of environmental microbiology.

## Experimental procedures

### Sampling procedure of forest soil

Forrest soil samples (*soil*) were taken from the ‘Nature Conservation Area’ Bergwerkswald at 50.564032 N 8.672555 E (Hasenkoeppel, Giessen, Germany). Five samples within a radius of ten metres (max. 10 cm in depth) including rhizosphere, lichen, humus soil and sandy loam were pooled. Samples were taken in December 2018 (Illumina approach) and January 2019 (cultivation campaign). Microorganisms were retrieved by Nycodenz density gradient centrifugation (Barra Caracciolo *et* *al*., 2005; Oberpaul *et* *al*., 2020). All samples were directly chilled and stored at 4°C until processing for the cultivation or at −50°C for Illumina amplicon sequencing.

### Encapsulation of microorganisms using microfluidic devices

The cell concentration of retrieved microorganisms was determined using the Bacteria Counting Kit (B7277, Invitrogen, Carlsbad, CA, USA) on a FACSCalibur (BD Bioscience, San Jose, CA, USA) according to the manufacturer’s protocol. The live/dead ratio was estimated using the LIVE/DEAD BacLight Bacterial Viability and Counting Kit (L7007, Invitrogen).

The commercially available µEncapsulator microfluidics set‐up, described in detail elsewhere (Caballero‐Aguilara *et* *al*., 2021), consisting of three pulse‐free pressure pumps (Mitos P‐Pump), a temperature control unit (TCU‐100), diverse microfluidic chips, polytetrafluoroethylene (PTFE) tubing (1/16″ OD, 0.25 mm ID and 0.5 mm ID) and connectors were purchased from Dolomite Microfluidics, a brand of Blacktrace Holdings Ltd (Royston, UK). A high‐speed CMOS camera PL‐D721CU (Navitar, Rochester, NY, USA) on a stereomicroscope Stemi SV 11 (Carl Zeiss, Oberkochen, Germany) equipped with a halogen light source KL 2500 LCD (Schott AG, Mainz, Germany) was used to image microfluidic operations. Samples for encapsulation were loaded onto the µEncapsulator Sample Reservoir Chip. Droplet generation was realized on a fluorophilic 50 µm µEncapsulator 2 Reagent Droplet Chip with an applied water to water to oil ratio of 1:1:20 at 30°C for ~ 30 min (Video S1). Pico‐Surf 1 (2% (w/w) in FC‐40) (Sphere Fluidics, Cambridge, UK) was used as the continuous oil phase. Both aqueous phases consisted of cultivation media with adjusted environmental or *E.coli* cell concentrations to obtain target cell distributions following Poisson (Fig. S1; Collins *et* *al*., 2015). All liquids except cell suspensions were filtered through a 0.2 µm CA syringe filter (Corning, Corning, NY, USA) to prevent blocking of the chip. Prior chip loading, media were mixed 50:50 with liquid, pre‐warmed 3% (w/v) SeaPlaque agarose (Lonza, Basel, Switzerland) in water. The droplets were collected in 1.5 ml reaction tubes and cooled at 4°C for 10 min to facilitate gelling of the agarose. Thereafter, droplets were incubated at 28°C in a humidity chamber for seven days for the Illumina amplicon sequencing and for the cultivation experiments. Neubauer chambers (0.1 mm depth, Paul Marienfeld GmbH KG, Lauda‐Königshofen, Germany) were used for imaging of stationary droplets on a fluorescence microscope DM2000 LED equipped with a DFC450 C camera and Las V4.7 software (Leica Microsystems, Wetzlar, Germany) for picture analysis.

### Cell staining and droplet sorting via FACS

Analysis and sorting of droplets were performed using a FACSCalibur. Surrounding oil was de‐emulsified upfront fluorescence staining and FACS analysis using Pico‐Break 1 (Dolomite Microfluidics) added in a ratio of 1:200 to a sample diluted with 1× PBS to adjust towards an analysis of 2000 events s^−1^. Fluorescence labelling of microorganisms was achieved by adding 30 µM DiOC_2_(3) (B34950, Invitrogen) to the samples and an incubation time of 5 min. Sorting via FACS (laser ex: 488 nm, em: 530 ± 30 nm [FL1‐H] and 670 ± 30 nm [FL3‐H]) was carried out using the single‐cell mode and 1× PBS as sheath fluid.

### Cultivation media

Media used for the growth of microorganisms in droplets, and the subsequent Illumina 16*S* V3‐V4 gene amplicon sequencing were as follows: VL55 supplemented with 0.05% (w/v) xylan (*VL55‐xyl*) or 0.05% (w/v) cellobiose (*VL55‐cello*), including selenite–tungstate solution and trace elements SL‐10 (Oberpaul *et* *al*., 2020) and buffered to pH 5.5. In addition, 1:10 diluted Reasoner’s 2A medium (*1:10* *R2A*, DMSZ medium 830), Minimal medium M9 (DMSZ medium 382), 1:20 diluted Casitone‐Yeast medium (*1:20* *CY*, DMSZ medium 67), 1:20 diluted Nutrient Broth (*1:20* *NB*, DMSZ medium 1), M13 with 200 mg l^−1^ ampicillin (*M13b*; (Wiegand *et* *al*., 2019)) and 1:20 diluted Tryptic Soy Broth medium (*1:20* *TSB*, Sigma‐Aldrich, St. Louis, MO, USA) were used.

For the cultivation campaign of microbial diversity using microfluidics and FACS, either VL55‐xyl or modified intensive soil extract medium (ISEM) (Nguyen *et* *al*., 2018) were used. ISEM was modified by addition of 1% (w/v) C‐source solution consisting of arabinogalactan, d‐(+)‐cellobiose, d‐(+)‐melezitose, xylan, galacto‐d‐mannan from *Ceratonia* *siliqua* and N‐acetylglucosamine (25 mg l^−1^ of each in deionized water) (m*ISEM^1^*). For the cultivation of sorted and arrayed events in 384‐well microplates (MTPs), we exchanged the soil extract with 0.1 g l^−1^ yeast extract (Oxoid) and 0.1 g l^−1^ casamino acids (Difco), 0.1 g l^−1^ proteose peptone (Roth, Karlsruhe, Germany) (*mISEM^2^*). Trace elements SL‐10 and selenite‐tungstate solution (each 2 ml l^−1^) were added to all media used for the cultivation experiments.

For the bioprospecting campaign, cultures were fermented in small‐scaled 96‐well Duetz system (Duetz *et* *al*., 2000) (Adolf Kühner AG, Birsfelden, Switzerland) using VL55‐xyl, ISP2 (DSMZ medium 987), basal salt medium (BSM) supplemented with glycerol (Marner *et* *al*., 2020) and mISEM^2^, adjusted to pH 5.5 and 7.2.

### Illumina amplicon sequencing and statistical analysis

In order to evaluate the relative cultivation success, we encapsulated the retrieved environmental microorganisms (λ0.1) considering the live/dead ratio in eight different media and incubated the droplets for seven days at 28°C. Environmental DNA was extracted using the NucleoSpin^®^ Soil DNA purification kit (Macherey Nagel, Düren, Germany) according to manufacturer’s protocol. The bacterial community composition of samples was assessed by Illumina 300‐bp paired‐end 16*S* V3‐V4 amplicon next‐generation sequencing using the degenerate primer pair 341F (3′‐CCTACGGGNGGCWGCAG‐5′) and 785R (3′‐GACTACHVGGGTATCTAAKCC‐5′). Sequencing was performed by LGC Genomics GmbH (Berlin, Germany) on a MiSeq (Illumina, San Diego, CA, USA), and data evaluation was supported by the SILVAngs pipeline (SILVA SSU Ref dataset; release 132; http://www.arb‐silva.de; SILVA Incremental Aligner (SINA SINA v1.2.10 for ARB SVN [revision 21008] Ribocon GmbH, Bremen, Germany)) and Blastn v2.2.30+ as previously described (Oberpaul *et* *al*., 2020). Phylogenetic groups with a relative abundance < 0.001% of total were excluded. Statistical calculations were done by using Past v4.03 (Hammer *et* *al*., 2001)) including the several‐sample one‐way ANOVA test on ranks (Daniel, 1990) followed by a Dunn’s post hoc test (Dunn, 1964) to judge on significances among samples.

### Microbial cultivation and extract preparation

Sorted droplets were concentrated using a 0.5 µm CA membrane filter (Whatman plc, Little Chalfont, UK) and a laboratory vacuum filtration system (Sartorius AG, Göttingen, Germany). Concentrated droplets were recovered in the respective growth medium (VL55‐xyl, mISEM^2^ pH 5.5 or pH 7.2) and arrayed into 384‐well MTPs with a distribution probability of *approx*. 0.25 droplets per well using a Matrix Wellmate microplate dispenser (Thermo Fisher Scientific, Waltham, MA, USA). After incubation as static cultures at 28°C in a humidity chamber for up to seven days, growth detection was assessed via turbidimetry (OD) at 600 nm using a Wallac 1420 Victor2 Microplate Reader (Perkin Elmer, Waltham, MA, USA) and by microscopy using a Zeiss Axiovert 200M (Carl Zeiss Microscopy GmbH, Jena, Germany) equipped with an SPOT RT Monochrome 2.1.1 camera (Diagnostic Instruments, Sterling Heights, MI, USA). All cultures exceeding our defined turbidity threshold (determined based on media control blanks present on each processed microplate) were automatically transferred into 96‐deep well MTPs pre‐filled with media (Corning, New York, NY, USA) using a Precision XS liquid handling system (BioTek Instruments GmbH, Bad Friedrichshall, Germany). These plates were incubated using the Duetz system at 28°C for seven days, shaking at 220 rpm, and 2.5 cm deflection. Sample aliquots were taken for DNA preparation (for 16*S* rRNA gene sequencing) and to generate a cryo‐conserved culture for long‐term conservation at −80°C.

For preparation of cryo‐conserved samples, 70% (v/v) glycerol and 5% (v/v) DMSO were filled into 96‐deep well MTPs and mixed with grown culture broths (ratio 2:3) using a VIAFLO 384 (Integra Biosciences, Biebertal, Germany).

Cryo‐conserved strains were recovered by agar plating and integrated into our strain collection. Bioprospecting campaigns were conducted either in 1 ml culture volume using 96‐well Duetz systems or in 50 ml culture volume using 300 ml Erlenmeyer flasks, respectively. Incubation of Erlenmeyer flasks occurred at 28°C using an RC‐406 orbital shaker (Infors, Bottmingen, Switzerland) with 5 cm deflection at 180 rpm for four and seven days. Fermentations were stopped by cooling microbial cultures and medium controls to −50°C. Frozen samples were lyophilized using a delta 2‐24 LSCplus (Martin Christ Gefriertrocknungsanlagen GmbH, Osterode am Harz, Germany), and an equal volume of methanol (related to the culture volume) was added. Extracts were further concentrated *in* *vacuo* (50‐fold in relation to the culture volume) for UHPLC‐QTOF‐MS/MS analysis. For screening purposes, extract aliquots were reconstituted in DMSO (100‐fold in relation to the culture broth).

### Phylogenetic classification and genotyping of isolated strains

DNA extraction was carried out by transferring 200 µl of each grown culture supernatant into Collection Microtubes (Qiagen, Hilden, Germany) containing three zirconia beads (2.3 mm, Carl Roth, Karlsruhe, Germany). Cells were disrupted (twice, 30 Hz for 1 min) by using a TissueLyser II (Qiagen). Plates were centrifuged at 4000 × *g* for 5 min, incubated at 70°C for 45 min and again centrifuged. The supernatants were transferred into fresh 96‐V‐bottom plates and used as template for 16*S* rRNA gene amplification following the PCR protocol described by (Kämpfer *et* *al*., 2014) using the primer pair E8F (5′‐GAGTTTGATCCTGGCTCAG‐3′) and 1492R (5′‐AGAGTTTGATCCTGGCTCAG‐3′) and for 18*S* rRNA gene amplification using the primer pair NS1 (5′‐GTAGTCATATGCTTGTCTC‐3′) and FR1 (5′‐AICCATTCAATCGGTAIT‐3′) following the protocol described by (Panzer *et* *al*., 2015). Genera belonging to Proteobacteria were defined as rarely cultured if less than ten representatives are recorded in the List of Prokaryotic names with Standing in Nomenclature (Parte, 2018, 2018; https://lpsn.dsmz.de/ access date: 01/2021).

All cultures affiliated to Acidobacteria were propagated on buffered R2A pH 5.5 agar to receive higher culture densities. All recovered nearly full‐length 16*S* and rRNA sequences were affiliated to the most similar sequences of type strains using Blastn (version: BLAST+ 2.11.0; http://blast.ncbi.nlm.nih.gov/Blast.cgi) with the NCBI Reference Sequence Database (version: RefSeq Release 202; https://www.ncbi.nlm.nih.gov/refseq/). All recovered nearly full‐length 18*S* rRNA sequences were affiliated to the most similar sequences of type strains using the pairwise alignment tool from MycoBank (https://www.mycobank.org/page/Pairwise_alignment). Representatives of different subgroups of Acidobacteria were included to visualize the phylogenetic relationship towards isolated Acidobacteria of this study. Therefore, multiple sequence alignment was done by Clustalw. Using the maximum‐likelihood method, a phylogenetic tree was calculated in Mega v7.0.26 (https://www.megasoftware.net) under the Tamura–Nei model (Kumar *et* *al*., 2016) performing 1000 bootstrap replications. Graphical modifications and annotations were made with itol v5.6.3 (https://itol.embl.de/; Letunic and Bork, 2019).

BOX‐A1R‐based repetitive extragenic palindromic sequence PCR (5′‐CTACGGCAAGGCGACGCTGACG‐3′) was used to amplify repetitive elements to perform molecular genotyping (Koeuth *et* *al*., 1995). Genomic fingerprinting patterns (BOX‐pattern) were analysed by LabChip GX Touch HT using DNA 5K Assay (Cat. No. CLS760675, PerkinElmer, Waltham, MA, USA) and Gelcompar II software version 6.5 (Applied Maths, Sint‐Martens‐Latem, Belgium) for data interpretation. A hierarchical cluster of the obtained data was calculated based on Dice similarity matrix data by applying Ward’s method (Ward, 1963) to discriminate genotypic redundancies within pure cultures.

### Bioassays

Crude extract screening was done by microplate broth dilution assays in final assay conc. 0.25‐fold, 0.5‐fold and onefold (in relation to the culture volume) against pathogenic bacteria and fungi (*Staphylococcus* *aureus* ATCC25923, *Mycobacterium* *smegmatis* ATCC 607, *Aspergillus* *flavus* ATCC 9170, *Zymoseptoria* campaign *ici* Roberge in Desmazières MUCL 45407 and *Candida* *albicans* FH 2173).

To evaluate the growth inhibitory effect of microbial extracts, a seeding cell suspension of the indicator strains was prepared from pre‐cultures or previously prepared spore solutions: for *S*. *aureus*, an overnight culture (37°C, 18 h, 180 rpm) was diluted to 2 × 10^4^ cells ml^−1^ in cation adjusted Mueller Hinton II medium (MHII). *M*. *smegmatis* was cultured in brain‐heart infusion medium (BD) supplemented with 1% (w/v) Tween‐80 (Sigma) for 48 h, before the assay cell concentration was adjusted (1 × 10^5^ cells ml^−1^). The pre‐culture of *C*. *albicans* was incubated for 48 h at 28°C before a 1 × 10^5^ cells ml^−1^ suspension was prepared in MHII. Spore solutions of *A*. *flavus* and *Z*. *tritici* were diluted to 1 × 10^5^ spores ml^−1^. A dilution series of gentamycin, isoniazid or nystatin was used as positive control and cell suspensions without extract or antibiotic as negative control. Test plates were incubated in the dark (37°C, 180 rpm, 85% rH) for 18 h (*A*. *flavus,* *S*. *aureus*), 48 h (*C*. *albicans,* *M*. *smegmatis*) and 72 h at 25°C (*Z*. *tritici*). End‐point detection towards optical density was determined using a LUMIstar^®^ Omega (BMG Labtech GmbH, Ortenberg, Germany) by measuring the turbidity at 600 nm or by ATP quantification using BacTiter‐Glo™ according to manufacturer’s protocol (Promega Corporation, Fitchburg, WI, USA).

### Mass spectrometry

All mass spectrometry experiments were performed on a 1290 UHPLC system (Agilent, Santa Clara, CA, USA) equipped with DAD, ELSD and maXis II™ (Bruker, Billerica, MA, USA) ESI‐QTOF‐UHRMS with the gradient: 0 min: 95% A; 0.30 min: 95% A; 18.00 min: 4.75% A; 18.10 min: 0% A; 22.50 min: 0% A; 22.60 min: 95% A; 25.00 min: 95% A (A: H_2_O, 0.1% formic acid (FA); B: acetonitrile, 0.1% FA; flow: 600 μl min^−1^). Column oven temperature: 45°C. Column: Acquity UPLC BEH C18 1.7 μm (2.1 × 100 mm) with Acquity UPLC BEH C18 1.7 μm VanGuard Pre‐Column (2.1 × 5 mm). Injection volume was either 1 or 2 µl.

### Cosine similarity calculations

Data processing was performed with Data Analysis 4.4 (Bruker, Billerica, MA, USA) using recalibration with sodium formate. *RecalculateLinespectra* with threshold 10 000 and subsequent *FindMolecularFeatures* (0.5–25 min, S/N = 0, correlation coefficient threshold = 0.7, minimum compound length = 8 spectra, smoothing width = 2) was performed. Bucketing was performed using *ProfileAnalysis* 2.3 (Bruker, Billerica, MA, USA) (30–1080 s, 100–1600 *m*/*z*, Advanced Bucketing with 12 s, 5 ppm, no transformation, Bucketing basis = H^+^). Samples with less than 50 features were excluded from further analysis. The generated bucket table was subsequently used as input for analysis *via* R (version 3.6.0) (R Core Team, 2020) with libraries readr (https://CRAN.R‐project.org/package=readr), coop (https://cran.r‐project.org/package=coop), gplots (https://CRAN.R‐project.org/package=gplots), data.table (https://CRAN.R‐project.org/package=data.table), and parallelDist (https://CRAN.R‐project.org/package=parallelDist). The cosine similarities (dot product of vectors) between samples were calculated. Samples were sorted according to clustering results, and pairwise similarities were extracted and subsequently used to define metabolic groups. The script used in this publication was deposited in a GitHub repository (Hartwig, 2020). If the pairwise similarity between two subsequent clustered samples is at the threshold 0.9 or higher, they were assigned to one metabolic group.

### Dereplication of bioactive extracts using UHPLC‐UHR MS/MS

Crude extracts showing at least 70% growth inhibition were considered as bioactive and were subjected to microfractionation. Five or ten microliter of concentrated methanolic extracts (50‐fold relative to the culture volume) were partitioned into 159 fractions (~ 7 s each) in a 384‐well MTP. UHPLC‐UHR‐MS analysis was performed (settings see Section Mass spectrometry). For microfractionation, 90% of the flow was collected with a custom made fraction collector (Zinsser–Analytic, Eschborn, Germany) while the rest was analysed in MS/MS mode in maXis II™. Collision induced dissociation was performed at 28.0–35.05 eV using argon at 10^−2^ mbar. Additionally to the chromatographically separated 159 fractions, crude extract was applied as fraction 160 in the same amount as the injection volume serving as positive control. All extracts were evaporated in a GeneVac HT‐12 (SP Industries Warminster, PA, USA) and were rescreened against the same test strain. Correlation between bioactive fractions and the corresponding MS data of analytes detected in those fractions was performed. Dereplication was facilitated by comparison of mass to charge ratios, retention time and fragmentation signatures with our in‐house reference database containing ~ 1700 structurally characterized microbial metabolites at the time of data processing. Molecular formula assignment was done manually for all compounds present in the active fractions, allowing a mass accuracy tolerance of ± 2 ppm. Annotation of the MS/MS spectra was performed manually for all the compounds present in active fractions, whenever no hits were found in the in‐house compound database. Molecular formula searches were performed on AntiBase 2017 (Laatsch, 2017) and Dictionary of Natural Products (http://dnp.chemnetbase.com/faces/chemical/ChemicalSearch.xhtml; accessed on Nov 16, 2020).

### Molecular networking

Based on published protocols, molecular networking with a cosine similarity cut‐off of > 0.7 was performed (Yang *et* *al*., 2013; Allard *et* *al*., 2016). The tool MSConvert (ProteoWizard package32) was used to convert the raw data (*.d files) into plain text files (*.mgf), wherein all detected fragment ions are expressed as a list of mass/intensity value pairs sorted according to their parent ions (peak picking: vendor MS level = 1–2; threshold type = absolute intensity, value = 1000, orientation = most‐intense). Sharing at least six fragments (tolerance Δppm 0.05) with at least one partner ion those ions were included in the final network (Riyanti *et* *al*., 2020). Known NPs were highlighted by including deposited compounds from the *in* *silico* fragmented (Allen *et* *al*., 2015) commercial database AntiBase 2017 (Laatsch, 2017) and our *in‐house* reference compound MS/MS database. The data were visualized with Cytoscape v3.6.0 (Shannon, 2003) as described elsewhere (Marner *et* *al*., 2020).

## Authors contributions

MO, SB, JG, and MS conceived and designed the experiments. MO, SB, MS, SM, BL, MM, CH, MP performed the experiments. MO, SB, MS, SM, MM, CH and MP analysed the data. MO, SB, MS and TFS drafted the first manuscript. TFS, JG and MS revised the manuscript. AV and PH initiated the public–private partnership between Fraunhofer and Sanofi (later Evotec). All authors accepted the final version of the manuscript.

## Conflict of interest

The authors declare no conflict of interest. The authors declare no competing financial interest.

## Supporting information


**Fig. S1.** Poisson distribution of λ0.1 and λ10 showing the probability of the amount of cells encapsulated per droplet.
**Fig. S2.** Work scheme of this study showing the microfluidics workflow, the subsequent cultivation, screening and dereplication
**Fig. S3.** Live/dead staining using the LIVE/DEAD BacLight Bacterial Viability and Counting Kit (L7007, Invitrogen). Manufacturer’s protocol was applied on the cells retrieved by nycodenz densitiy gradient centrifugation (A: SYTO 9, B: propidium iodide, C: merged) directly after bacterial isolation. Exemplary pictures are shown, ten independent stains were done and considered for the calculation. Live:dead ratio was estimated resulting in ~ 70:30 ± 6.7%.
**Fig. S4.** Phylogenetic classification of FHG110511 within the phylum Acidobacteria clustering into subgroup 1. The tree is based on a ClustalW alignment of available 16*S* rRNA gene sequences from the ref_seq database between positions 113 and 1357 [based on Escherichia coli 16S rRNA gene numbering (Brosius et al., 1978)] from the most similar sequences to the isolated strains, and also includes representatives of Acidobacteria subgroups 1, 3, 4, 6, 7, 8, and 10. The tree was calculated using Mega v7.0.26 with the maximum‐likelihood method and GTR‐Gamma model. Circles on the tree branches indicate values of 1000 bootstrap replicates with a bootstrap support of more than 50%. Subgroup affiliations are indicated by colors. The new isolate is indicated by a black arrow. The tree is drawn to scale, with branch lengths measured in the number of substitutions per site.
**Fig. S5.** (A) Assay read‐out of μ‐fractionation plates of strain FHG110488 against *M*. *smegmatis* ATCC 607. Fractions are numbered and those causing at least 70% rel. growth inhibition were considered “active” and marked red. Column 1: medium control; Column 2+3: antibiotic standard (isoniazid); Column 4: growth control. Area AH05‐AH24 top: 5 μl injection volume; Area AH05‐AH24 bottom: 10 μl injection; Crude: crude extract as a control. (B) Overlaid Base peak Chromatogram (grey), fraction collector analog signal (light blue bars) and extracted ion chromatogram of *m*/*z* 515.3329±0.005 [M+H]^+^ (**1**, red) of the 50 fold concentrated extract (in MeOH) with 5 μl injection volume. (C) UV and MS spectrum of fractions 84‐87. (D) MS/MS fragmentation of the precursor ion at *m*/*z* 515.3330 [M+H]^+^ (dereplicated as Serratamolide A, displayed in red) with manual annotation of the neutral losses.
**Fig. S6.** (A) MS2‐network of “active” extract of FHG110488 against *Septoria* *tritici* MUCL45407 focusing on the cluster representing all seven detected serratamolide derivatives and their literature known structures (dots of parent ions found as hits in our internal database or AntiBase are marked in gold). (B) Overlaid Base peak Chromatogram (grey) and extracted ion chromatograms of serratamolides **1–7** (**1** *m*/*z* 515.3327 [M+H]^+^, C_26_H_47_N_2_O_8_^+^ (red); **2** *m*/*z* 541.3483 [M+H]^+^, C_28_H_49_N_2_O_8_^+^ (blue); **3** *m*/*z* 543.3640 [M+H]^+^, C_28_H_51_N_2_O_8_^+^ (black); **4** *m*/*z* 533.3433 [M+H]^+^, C_26_H_49_N_2_O_9_^+^ (cyan); **5** *m*/*z* 547.3598 [M+H]^+^, C_27_H_51_N_2_O_9_^+^ (yellow); **6** *m*/*z* 575.3902 [M+H]^+^, C_29_H_55_N_2_O_9_^+^ (light green); **7** *m*/*z* 573.3746 [M+H]^+^, C_29_H_53_N_2_O_9_^+^ (dark green)) of the 50 fold concentrated extract (in MeOH) with 5 μL injection volume. (C) MS/MS fragmentation of the precursor ions **1**‐**7**.
**Fig. S7.** (A) Assay read‐out of μ‐fractionation plate of strain FHG110502 against *Mycobacterium* *smegmatis* ATCC 607. Fractions are numbered and those causing at least 70% rel. growth inhibition were considered “active” and marked red. Column 1: medium control; Column 2+3: antibiotic standard (isoniazid); Column 4: growth control. Area AH05‐AH24: 2 μl injection volume; Area IP05‐IP24: 5 μl injection; Crude: crude extract as a control. (B) Overlaid Base peak Chromatograms (grey), Fraction collector analog signals (light blue bars) and extracted ion chromatogram s of *m*/*z* 1112.6814±0.005 [M+H]^+^ (**8**, red) with corresponding *m*/*z* 556.8446±0.005 [M+2H]^2+^ (green), *m*/*z* 1126.6973±0.005 [M+H]^+^ (**9**, yellow) with corresponding *m*/*z* 563.8524 ± 0.005 [M+2H]^2+^ (blue), and *m*/*z* 1154.7288±0.005 [M+H]^+^ (**10**, purple) with corresponding *m*/*z* 577.8680±0.005 [M+2H]^2+^ (magenta) of the 50 fold concentrated extract (in MeOH) with 5 μl injection volume. (C) UV and MS spectrum of fractions 105–106 (left), 108–118 (middle) and 116 (right). (D) MS/MS fragmentation of the precursor ion at *m*/*z* 1112.6814 [M+H]^+^, *m*/*z* 1126.6973 [M+H]^+^, and *m*/*z* 1154.7288 [M+H]^+^ (dereplicated as massetolide E, massetolide F and massetolide H, respectively), manual annotation of the neutral losses and proposed structures of the fragment ions at *m*/*z* 284.2229 and *m*/*z* 312.2533. (E) Structures of all three dereplicated compounds **8–10**.
**Fig. S8.** (A) Assay read‐out of μ‐fractionation plate of strain FHG110502 against *Mycobacterium* *smegmatis* ATCC 607. Fractions are numbered and those causing at least 70% rel. growth inhibition were considered “active” and marked red. Column 1: medium control; Column 2+3: antibiotic standard (isoniazid); Column 4: growth control. Area AH05‐AH24: 2 μl injection volume; Area IP05‐IP24: 5 μl injection; Crude: crude extract as a control. (B) Overlaid Base peak Chromatograms (grey), Fraction collector analog signals (light blue bars) and extracted ion chromatogram s of *m*/*z* 454.2931 ± 0.005 [M+H]^+^ (**11**, red), *m*/*z* 690.5073 ± 0.005 [M+H]^+^ (**12**, blue) and *m*/*z* 716.5237 ± 0.005 [M+H]^+^ (**13**, green)of the 50 fold concentrated extract (in MeOH) with 5 μl injection volume. (C) UV and MS spectrum of fractions 93–94 (left) and 130–136 (right). (D) MS/MS fragmentation of the precursor ion at *m*/*z* 454.2931 [M+H]^+^, *m*/*z* 690.5073 [M+H]^+^, and *m*/*z* 716.5237 [M+H]^+^ (dereplicated as lyso‐palmitoyl‐phosphoethanolamine, palmitoleoyl‐palmitoyl‐phosphoetanolamine and palmitoleoyl‐oleoyl‐phosphoetanolamine, respectively), manual annotation of the neutral losses and proposed structures of the fragment ions. (E) Putative structures of all three dereplicated compounds **11–13**.
**Fig. S9.** (A) Assay read‐out of μ‐fractionation plate of strain FHG110508 against *Staphylococcus* *aureus* ATCC 25923. Fractions are numbered and those causing at least 70% rel. growth inhibition were considered “active” and marked red. Column 1: medium control; Column 2+3: antibiotic standard (gentamycin); Column 4: growth control. Area AH05‐AH24: 2 μl injection volume; Area IP05‐IP24: 5 μl injection; Crude: crude extract as a control. (B) Overlaid Base peak Chromatograms (grey), Fraction collector analog signals (light blue bars) and extracted ion chromatogram s of *m*/*z* 737.4475±0.005 [M+H]^+^ (**14**, red), *m*/*z* 751.4636±0.005 [M+H]^+^ (**15**, green), *m*/*z* 765.4794±0.005 [M+H]^+^ (**16**, blue), and *m*/*z* 779.4956±0.005 [M+H]^+^ (**17**, yellow) of the 50 fold concentrated extract (in MeOH) with 5 μl injection volume. (C) UV and MS spectrum of fractions 120, 124, 128 and 131‐132 (from left to right). (D) MS/MS fragmentation of the precursor ion at *m*/*z* 737.4462 [M+H]^+^, *m*/*z* 751.4618 [M+H]^+^, *m*/*z* 765.4779 [M+H]^+^, and *m*/*z* 779.4933 [M+H]^+^ (dereplicated as nonactin, monactin, dinactin and macrotetrolide G, respectively) with manual annotation of the neutral loss and proposed structure of the fragment ion at *m*/*z* 213.1482 of parent ion at *m*/*z* 779.4933 indicating the presents of macrotetrolide G instead of trinactin. (E) Structures of all four dereplicated macrotetrolides. Me: methyl; Et: ethyl; iPr: isopropyl.
**Fig. S10.** (A) MS2‐network of “active” extract of FHG110508 against *Staphylococcus* *aureus* ATCC 25923 with focus on the cluster representing all five detected macrotetrolide derivatives and their adduct ions (dots of parent ions found as hits in our internal database or AntiBase are marked in gold). (B) Overlaid Base peak Chromatogram (grey) and extracted ion chromatograms of macrotetrolide **14**‐**18** (**14** *m*/*z* 737.4462 [M+H]^+^, C_40_H_65_O_12_^+^ (red); **15** *m*/*z* 751.4618 [M+H]^+^, C_41_H_67_O_12_^+^ (green); **16** *m*/*z* 765.4779 [M+H]^+^, C_42_H_69_O_12_^+^ (blue); **17** *m*/*z* 779.4933 [M+H]^+^, C_43_H_71_O_12_^+^ (yellow); **18** *m*/*z* 793.5104 [M+H]^+^, C_44_H_73_O_12_^+^ (black)) of the 50 fold concentrated extract (in MeOH) with 5 μl injection volume. (C) MS/MS fragmentation of the precursor ions **14–18** dereplicated as nonactin, monactin, dinactin, macrotetrolide G, and macrotetrolide D, respectively, with manual annotation of the neutral loss and proposed structure of the fragment ion at *m*/*z* 213.1482 of parent ion at *m*/*z* 779.4933 indicating the presents of macrotetrolide G instead of trinactin and fragment ion at *m*/*z* 213.1487 of parent ion at *m*/*z* 793.5104 indicating the presents of macrotetrolide D instead of tetranactin.
**Table S1.** Overview of the cultured genera in mISEM^2^ pH 7.2, pH 5.5 and VL55‐xyl pH 5.5. 16*S* rRNA gene sequencing was applied using the primer 1492R on mechanically disrupted culture broth grown for 7 days.
**Table S2.** Cosine Similarity table – Data for Fig. affiliation.

**Video S1.** Video showing the production of droplets with the µEncapsulator from Dolomite.

## Data Availability

The Illumina amplicon sequencing data supporting the findings of this study are openly available under the BioProject PRJNA699730 (https://www.ncbi.nlm.nih.gov/bioproject/PRJNA699730).

## References

[mbt213872-bib-0001] Adamek, M. , Alanjary, M. , and Ziemert, N. (2019) Applied evolution: phylogeny‐based approaches in natural products research. Nat Prod Rep 36: 1295–1312. 31475269 10.1039/c9np00027e

[mbt213872-bib-0002] Akselband, Y. , Cabral, C. , Castor, T.P. , Chikarmane, H.M. , and McGrath, P. (2006) Enrichment of slow‐growing marine microorganisms from mixed cultures using gel microdrop (GMD) growth assay and fluorescence‐activated cell sorting. J Exp Mar Biol Ecol 329: 196–205.

[mbt213872-bib-0003] Allard, P.‐M. , Péresse, T. , Bisson, J. , Gindro, K. , Marcourt, L. , van Pham, C. , *et* *al*. (2016) Integration of molecular networking and in‐silico MS/MS fragmentation for natural products dereplication. Anal Chem 88: 3317–3323. 26882108 10.1021/acs.analchem.5b04804

[mbt213872-bib-0004] Allen, F. , Greiner, R. , and Wishart, D. (2015) Competitive fragmentation modeling of ESI‐MS/MS spectra for putative metabolite identificatio. Metabolomics 11: 98–110.

[mbt213872-bib-0005] Baret, J.‐C. , Miller, O.J. , Taly, V. , Ryckelynck, M. , El‐Harrak, A. , Frenz, L. , *et* *al*. (2009) Fluorescence‐activated droplet sorting (FADS): efficient microfluidic cell sorting based on enzymatic activity. Lab Chip 9: 1850. 19532959 10.1039/b902504a

[mbt213872-bib-0006] Barra Caracciolo, A. , Grenni, P. , Cupo, C. , and Rossetti, S. (2005) In situ analysis of native microbial communities in complex samples with high particulate loads. FEMS Microbiol Lett 253: 55–58. 16213678 10.1016/j.femsle.2005.09.018

[mbt213872-bib-0007] Belknap, K.C. , Park, C.J. , Barth, B.M. , and Andam, C.P. (2020) Genome mining of biosynthetic and chemotherapeutic gene clusters in Streptomyces bacteria. Sci Rep 10: 1–9. 32029878 10.1038/s41598-020-58904-9 PMC7005152

[mbt213872-bib-0008] Biener, G. , Masson‐Meyers, D.S. , Bumah, V.V. , Hussey, G. , Stoneman, M.R. , Enwemeka, C.S. , and Raicu, V. (2017) Blue/violet laser inactivates methicillin‐resistant Staphylococcus aureus by altering its transmembrane potential. J Photochem Photobiol B Biol 170: 118–124. 10.1016/j.jphotobiol.2017.04.002 28426977

[mbt213872-bib-0009] Boedicker, J.Q. , Vincent, M.E. , and Ismagilov, R.F. (2009) Microfluidic confinement of single cells of bacteria in small volumes initiates high‐density behavior of quorum sensing and growth and reveals its variability. Angew Chem Int Ed Engl 48: 5908–5911. 19565587 10.1002/anie.200901550 PMC2748941

[mbt213872-bib-0010] Boitard, L. , Cottinet, D. , Bremond, N. , Baudry, J. , and Bibette, J. (2015) Growing microbes in millifluidic droplets. Eng Life Sci 15: 318–326.

[mbt213872-bib-0011] Bon, R.S. , and Waldmann, H. (2010) Bioactivity‐guided navigation of chemical space. Acc Chem Res 43: 1103–1114. 20481515 10.1021/ar100014h

[mbt213872-bib-0012] Butler, M.S. , and Paterson, D.L. (2020) Antibiotics in the clinical pipeline in October 2019. J Antibiot 73: 329–364. 10.1038/s41429-020-0291-8 PMC7223789 32152527

[mbt213872-bib-0013] Caballero‐Aguilara, L.M. , Duchib, S. , Quigleyd, A. , Onofrillob, C. , Di Bellab, C. , and Moultona, S.E. (2021) Microencapsulation of growth factors by microfluidic system. MethodsX 8: 101324. 34434839 10.1016/j.mex.2021.101324 PMC8374335

[mbt213872-bib-0014] Collins, D.J. , Neild, A. , deMello, A. , Liu, A.Q. , and Ai, Y. (2015) The Poisson distribution and beyond: methods for microfluidic droplet production and single cell encapsulation. Lab Chip 15: 3439–3459. 26226550 10.1039/c5lc00614g

[mbt213872-bib-0015] Crits‐Christoph, A. , Diamond, S. , Butterfield, C.N. , Thomas, B.C. , and Banfield, J.F. (2018) Novel soil bacteria possess diverse genes for secondary metabolite biosynthesis. Nature 558: 440–444. 29899444 10.1038/s41586-018-0207-y

[mbt213872-bib-0016] Damsté, J.S.S. , Rijpstra, W.I.C. , Dedysh, S.N. , Foesel, B.U. , and Villanueva, L. (2017) Pheno‐ and Genotyping of Hopanoid Production in Acidobacteria. Front Microbiol 8: 968. 28642737 10.3389/fmicb.2017.00968 PMC5462960

[mbt213872-bib-0017] Daniel, W.W. (1990) Applied nonparametric statistics. PWS‐KENT Pub. University of Michigan. URL https://books.google.de/books?id=0hPvAAAAMAAJ.

[mbt213872-bib-0018] Delgado‐Baquerizo, M. , Maestre, F.T. , Reich, P.B. , Jeffries, T.C. , Gaitan, J.J. , Encinar, D. , *et* *al*. (2016) Microbial diversity drives multifunctionality in terrestrial ecosystems. Nat Commun 7: 10541. 26817514 10.1038/ncomms10541 PMC4738359

[mbt213872-bib-0019] Duetz, W.A. , Rüedi, L. , Hermann, R. , O'Connor, K. , Büchs, J. , and Witholt, B. (2000) Methods for intense aeration, growth, storage, and replication of bacterial strains in microtiter plates. Appl Environ Microbiol 66: 2641–2646. 10831450 10.1128/aem.66.6.2641-2646.2000 PMC110593

[mbt213872-bib-0020] Dunn, O.J. (1964) Multiple comparisons using rank sums. Technometrics 6: 241–252.

[mbt213872-bib-0021] Dwivedi, D. , Jansen, R. , Molinari, G. , Nimtz, M. , Johri, B.N. , and Wray, V. (2008) Antimycobacterial serratamolides and diacyl peptoglucosamine derivatives from Serratia sp. J Nat Prod 71: 637–641. 18303848 10.1021/np7007126

[mbt213872-bib-0022] Firn, R.D. , and Jones, C.G. (2003) Natural products ‐ a simple model to explain chemical diversity. Nat Prod Rep 20: 382–391. 12964834 10.1039/b208815k

[mbt213872-bib-0023] Fox, J.L. (2014) Fraunhofer to mine Sanofi microbial collection. Nat Biotechnol 32: 305. 24714464 10.1038/nbt0414-305a

[mbt213872-bib-0024] Gerard, J. , Lloyd, R. , Barsby, T. , Haden, P. , Kelly, M.T. , and Andersen, R.J. (1997) Massetolides A‐H, antimycobacterial cyclic depsipeptides produced by two Pseudomonads isolated from marine habitats. J Nat Prod 60: 223–229. 9157190 10.1021/np9606456

[mbt213872-bib-0025] Gross, H. , and Loper, J.E. (2009) Genomics of secondary metabolite production by Pseudomonas spp. Nat Prod Rep 26: 1408. 19844639 10.1039/b817075b

[mbt213872-bib-0026] Hammer, Ø. , Harper, D.A.T. , and Ryan, P.D. (2001) PAST: Paleontologocal statistics software package for education and data analysis. Palaeontol Electron 4: 1–9.

[mbt213872-bib-0027] Hartwig, C. (2020) christoph‐hartwig‐ime‐br/cosine‐V2: cosine‐v2. Zenodo.

[mbt213872-bib-0028] Hoffmann, T. , Krug, D. , Bozkurt, N. , Duddela, S. , Jansen, R. , Garcia, R. , *et* *al*. (2018) Correlating chemical diversity with taxonomic distance for discovery of natural products in myxobacteria. Nat Commun 9: 803. 29476047 10.1038/s41467-018-03184-1 PMC5824889

[mbt213872-bib-0029] Hong, J. (2011) Role of natural product diversity in chemical biology. Curr Opin Chem Biol 15: 350–354. 21489856 10.1016/j.cbpa.2011.03.004 PMC3110584

[mbt213872-bib-0030] Imai, Y.u. , Meyer, K.J. , Iinishi, A. , Favre‐Godal, Q. , Green, R. , Manuse, S. , *et* *al*. (2019) A new antibiotic selectively kills Gram‐negative pathogens. Nature 576: 459–464. 31747680 10.1038/s41586-019-1791-1 PMC7188312

[mbt213872-bib-0031] Ishii, S. , Tago, K. , and Senoo, K. (2010) Single‐cell analysis and isolation for microbiology and biotechnology: methods and applications. Appl Microbiol Biotechnol 86: 1281–1292. 20309540 10.1007/s00253-010-2524-4

[mbt213872-bib-0032] Kaeberlein, T. (2002) Isolating “Uncultivable” microorganisms in pure culture in a simulated natural environment. Science 296: 1127–1129. 12004133 10.1126/science.1070633

[mbt213872-bib-0033] Kämpfer, P. , Dott, W. , Martin, K. , and Glaeser, S.P. (2014) Rhodococcus defluvii sp. nov., isolated from wastewater of a bioreactor and formal proposal to reclassify [*Corynebacterium* *hoagii*] and Rhodococcus equi as Rhodococcus hoagii comb. nov. Int J Syst Evol Microbiol 64(Pt 3): 755–761. 24198057 10.1099/ijs.0.053322-0

[mbt213872-bib-0034] Keller, M. , and Zengler, K. (2004) Tapping into microbial diversity. Nat Rev Microbiol 2: 141–150. 15040261 10.1038/nrmicro819

[mbt213872-bib-0035] Kielak, A.M. , Barreto, C.C. , Kowalchuk, G.A. , van Veen, J.A. , and Kuramae, E.E. (2016) The ecology of Acidobacteria: moving beyond genes and genomes. Front Microbiol 7: 744. 27303369 10.3389/fmicb.2016.00744 PMC4885859

[mbt213872-bib-0036] Koch, I.H. , Gich, F. , Dunfield, P.F. , and Overmann, J. (2008) Edaphobacter modestus gen. nov., sp. nov., and Edaphobacter aggregans sp. nov., acidobacteria isolated from alpine and forest soils. Int J Syst Evol Microbiol 58: 1114–1122. 18450699 10.1099/ijs.0.65303-0

[mbt213872-bib-0037] Koeuth, T. , Versalovic, J. , and Lupski, J.R. (1995) Differential subsequence conservation of interspersed repetitive *Streptococcus* *pneumoniae* BOX elements in diverse bacteria. Genome Res 5: 408–418. 8750201 10.1101/gr.5.4.408

[mbt213872-bib-0038] Kumar, S. , Stecher, G. , and Tamura, K. (2016) MEGA7: Molecular Evolutionary Genetics Analysis Version 7.0 for Bigger Datasets. Mol Biol Evol 33: 1870–1874. 27004904 10.1093/molbev/msw054 PMC8210823

[mbt213872-bib-0039] Laatsch, H. (2017) AntiBase: The Natural Compound Identifier. Weinheim, Germany: Wiley‐VCH.

[mbt213872-bib-0040] Lakemeyer, M. , Zhao, W. , Mandl, F.A. , Hammann, P. , and Sieber, S.A. (2018) Über bisherige Denkweisen hinaus ‐ neue Wirkstoffe zur Überwindung der Antibiotika‐Krise. Angew Chem 130: 14642–14682.

[mbt213872-bib-0041] Leman, M. , Abouakil, F. , Griffiths, A.D. , and Tabeling, P. (2015) Droplet‐based microfluidics at the femtolitre scale. Lab Chip 15: 753–765. 25428861 10.1039/c4lc01122h

[mbt213872-bib-0042] Letunic, I. , and Bork, P. (2019) Interactive Tree Of Life (iTOL) v4: recent updates and new developments. Nucleic Acids Res 47: W256–W259. 30931475 10.1093/nar/gkz239 PMC6602468

[mbt213872-bib-0043] Lewis, K. (2013) Platforms for antibiotic discovery. Nat Rev Drug Discov 12: 371–387. 23629505 10.1038/nrd3975

[mbt213872-bib-0044] Ling, L.L. , Schneider, T. , Peoples, A.J. , Spoering, A.L. , Engels, I. , Conlon, B.P. , *et* *al*. (2015) A new antibiotic kills pathogens without detectable resistance. Nature 517: 455–459. 25561178 10.1038/nature14098 PMC7414797

[mbt213872-bib-0045] Lok, C. (2015) Mining the microbial dark matter. Nature 522: 270–273. 26085253 10.1038/522270a

[mbt213872-bib-0046] Mahler, L. , Tovar, M. , Weber, T. , Brandes, S. , Rudolph, M.M. , Ehgartner, J. , *et* *al*. (2015) Enhanced and homogeneous oxygen availability during incubation of microfluidic droplets. RSC Adv 5: 101871–101878.

[mbt213872-bib-0047] Marner, M. , Patras, M.A. , Kurz, M. , Zubeil, F. , Förster, F. , Schuler, S. , *et* *al*. (2020) Molecular networking‐guided discovery and characterization of stechlisins, a group of cyclic lipopeptides from a Pseudomonas sp. J Nat Prod 83: 2607–2617. 32822175 10.1021/acs.jnatprod.0c00263

[mbt213872-bib-0048] Medema, M.H. , Cimermancic, P. , Sali, A. , Takano, E. , and Fischbach, M.A. (2014) A systematic computational analysis of biosynthetic gene cluster evolution: lessons for engineering biosynthesis. PLoS Comput Biol 10: e1004016. 25474254 10.1371/journal.pcbi.1004016 PMC4256081

[mbt213872-bib-0049] Monciardini, P. , Iorio, M. , Maffioli, S. , Sosio, M. , and Donadio, S. (2014) Discovering new bioactive molecules from microbial sources. Microb Biotechnol 7: 209–220. 24661414 10.1111/1751-7915.12123 PMC3992017

[mbt213872-bib-0050] Mulligan, C.N. (2005) Environmental applications for biosurfactants. Environ Pollut 133: 183–198. 15519450 10.1016/j.envpol.2004.06.009

[mbt213872-bib-0051] Newman, D.J. , and Cragg, G.M. (2020) Natural products as sources of new drugs over the nearly four decades from 01/1981 to 09/2019. J Nat Prod 83: 770–803. 32162523 10.1021/acs.jnatprod.9b01285

[mbt213872-bib-0052] Nge, P.N. , Rogers, C.I. , and Woolley, A.T. (2013) Advances in microfluidic materials, functions, integration, and applications. Chem Rev 113: 2550–2583. 23410114 10.1021/cr300337x PMC3624029

[mbt213872-bib-0053] Nguyen, T.M. , Seo, C. , Ji, M. , Paik, M.‐J. , Myung, S.‐W. , and Kim, J. (2018) Effective soil extraction method for cultivating previously uncultured soil bacteria. Appl Environ Microbiol 84: e01145‐18. 30291118 10.1128/AEM.01145-18 PMC6275337

[mbt213872-bib-0054] Nicault, M. , Tidjani, A.‐R. , Gauthier, A. , Dumarcay, S. , Gelhaye, E. , Bontemps, C. , and Leblond, P. (2020) Mining the biosynthetic potential for specialized metabolism of a Streptomyces soil community. Antibiotics 9: 271. 10.3390/antibiotics9050271 PMC7277575 32456220

[mbt213872-bib-0055] Oberpaul, M. , Zumkeller, C.M. , Culver, T. , Spohn, M. , Mihajlovic, S. , Leis, B. , *et* *al*. (2020) High‐throughput cultivation for the selective isolation of Acidobacteria from termite nests. Front Microbiol 11: 597628. 33240253 10.3389/fmicb.2020.597628 PMC7677567

[mbt213872-bib-0056] Overmann, J. , Abt, B. , and Sikorski, J. (2017) Present and future of culturing bacteria. Annu Rev Microbiol 71: 711–730. 28731846 10.1146/annurev-micro-090816-093449

[mbt213872-bib-0057] Owen, W.J. , Yao, C. , Myung, K. , Kemmitt, G. , Leader, A. , Meyer, K.G. , *et* *al*. (2017) Biological characterization of fenpicoxamid, a new fungicide with utility in cereals and other crops. Pest Manag Sci 73: 2005–2016. 28471527 10.1002/ps.4588 PMC5599960

[mbt213872-bib-0058] Panthee, S. , Hamamoto, H. , Paudel, A. , and Sekimizu, K. (2016) Lysobacter species: a potential source of novel antibiotics. Arch Microbiol 198: 839–845. 27541998 10.1007/s00203-016-1278-5

[mbt213872-bib-0059] Panzer, K. , Yilmaz, P. , Weiß, M. , Reich, L. , Richter, M. , Wiese, J. , *et* *al*. (2015) Identification of habitat‐specific biomes of aquatic fungal communities using a comprehensive nearly full‐length 18S rRNA dataset enriched with contextual data. PLoS One 10: e0134377. 26226014 10.1371/journal.pone.0134377 PMC4520555

[mbt213872-bib-0060] Parte, A.C. (2018) LPSN ‐ List of Prokaryotic names with Standing in Nomenclature (bacterio.net), 20 years on. Int J Syst Evol Microbiol 68: 1825–1829. 29724269 10.1099/ijsem.0.002786

[mbt213872-bib-0061] Payne, E.M. , Holland‐Moritz, D.A. , Sun, S. , and Kennedy, R.T. (2020) High‐throughput screening by droplet microfluidics: perspective into key challenges and future prospects. Lab Chip 20: 2247–2262. 32500896 10.1039/d0lc00347f PMC12183681

[mbt213872-bib-0062] Phillies, G. (1975) Nonactin, monactin, dinactin, trinactin, and tetranactin. A Raman Spectroscopic Study. Biopolymers 14: 2311–2327. 10.1002/bip.1977.360160112 576586

[mbt213872-bib-0063] Pöppel, A.‐K. , Koch, A. , Kogel, K.‐H. , Vogel, H. , Kollewe, C. , Wiesner, J. , and Vilcinskas, A. (2014) Lucimycin, an antifungal peptide from the therapeutic maggot of the common green bottle fly Lucilia sericata. Biol Chem 395: 649–656. 24622788 10.1515/hsz-2013-0263

[mbt213872-bib-0064] R Core Team (2020) R: A Language and Environment for Statistical Computing. Vienna, Austria: R Foundation for Statistical Computing.

[mbt213872-bib-0065] Řezanka, T. , Prell, A. , Spížek, J. , and Sigler, K. (2010) Pilot‐plant cultivation of Streptomyces griseus producing homologues of nonactin by precursor‐directed biosynthesis and their identification by LC/MS‐ESI. J Antibiot 63: 524–529. 10.1038/ja.2010.93 20664602

[mbt213872-bib-0066] Riyanti , Marner, M. , Hartwig, C. , Patras, M. , Wodi, S. , Rieuwpassa, F. , *et* *al*. (2020) Sustainable low‐volume analysis of environmental samples by Semi‐Automated Prioritization of Extracts for Natural Product Research (SeaPEPR). Mar Drugs 18: 649. 10.3390/md18120649 PMC7765863 33348536

[mbt213872-bib-0067] Schäberle, T.F. , and Hack, I.M. (2014) Overcoming the current deadlock in antibiotic research. Trends Microbiol 22: 165–167. 24698433 10.1016/j.tim.2013.12.007

[mbt213872-bib-0068] Shannon, P. (2003) Cytoscape: A software environment for integrated models of biomolecular interaction networks. Genome Res 13: 2498–2504. 14597658 10.1101/gr.1239303 PMC403769

[mbt213872-bib-0069] Shapiro, H.M. (2000) Membrane potential estimation by flow cytometry. Methods 21: 271–279. 10873481 10.1006/meth.2000.1007

[mbt213872-bib-0070] Stewart, E.J. (2012) Growing unculturable bacteria. J Bacteriol 194: 4151–4160. 22661685 10.1128/JB.00345-12 PMC3416243

[mbt213872-bib-0071] Strobel, G.A. , Morrison, S.L. , and Cassella, M. (2005) Protecting plants from oomycete pathogens by treatment with compositions containing serratamolide and oocydin a from Serratia marcescens. Patent No.: US6926892B2.

[mbt213872-bib-0072] Tacconelli, E. , Carrara, E. , Savoldi, A. , Harbarth, S. , Mendelson, M. , Monnet, D.L. , *et* *al*. (2018) Discovery, research, and development of new antibiotics: the WHO priority list of antibiotic‐resistant bacteria and tuberculosis. Lancet Infect Dis 18: 318–327. 29276051 10.1016/S1473-3099(17)30753-3

[mbt213872-bib-0073] Tamehiro, N. , Okamoto‐Hosoya, Y. , Okamoto, S. , Ubukata, M. , Hamada, M. , Naganawa, H. , and Ochi, K. (2002) Bacilysocin, a Novel phospholipid antibiotic produced by Bacillus subtilis 168. Antimicrob Agents Chemother 46: 315–320. 11796336 10.1128/AAC.46.2.315-320.2002 PMC127064

[mbt213872-bib-0074] Terekhov, S.S. , Smirnov, I.V. , Stepanova, A.V. , Bobik, T.V. , Mokrushina, Y.A. , Ponomarenko, N.A. , *et* *al*. (2017) Microfluidic droplet platform for ultrahigh‐throughput single‐cell screening of biodiversity. Proc Natl Acad Sci USA 114: 2550–2555. 28202731 10.1073/pnas.1621226114 PMC5347554

[mbt213872-bib-0075] Theberge, A.B. , Courtois, F. , Schaerli, Y. , Fischlechner, M. , Abell, C. , Hollfelder, F. , and Huck, W.T. (2010) Microdroplets in microfluidics: an evolving platform for discoveries in chemistry and biology. Angew Chem Int Ed Engl 49: 5846–5868. 20572214 10.1002/anie.200906653

[mbt213872-bib-0076] Theuretzbacher, U. , Outterson, K. , Engel, A. , and Karlén, A. (2020) The global preclinical antibacterial pipeline. Nat Rev Microbiol 18: 275–285. 31745331 10.1038/s41579-019-0288-0 PMC7223541

[mbt213872-bib-0077] Thies, S. , Santiago‐Schübel, B. , Kovačić, F. , Rosenau, F. , Hausmann, R. , and Jaeger, K.‐E. (2014) Heterologous production of the lipopeptide biosurfactant serrawettin W1 in Escherichia coli. J Biotechnol 181: 27–30. 24732103 10.1016/j.jbiotec.2014.03.037

[mbt213872-bib-0078] Tovar, M. , Mahler, L. , Buchheim, S. , Roth, M. , and Rosenbaum, M.A. (2020) Monitoring and external control of pH in microfluidic droplets during microbial culturing. Microb Cell Fact 19: 16. 31996234 10.1186/s12934-020-1282-y PMC6990587

[mbt213872-bib-0079] Tracanna, V. , de Jong, A. , Medema, M.H. , and Kuipers, O.P. (2017) Mining prokaryotes for antimicrobial compounds: from diversity to function. FEMS Microbiol Rev 41: 417–429. 28402441 10.1093/femsre/fux014

[mbt213872-bib-0080] Umetsu, N. , and Shirai, Y. (2020) Development of novel pesticides in the 21st century. J Pestic Sci 45: 54–74. 33132734 10.1584/jpestics.D20-201 PMC7581488

[mbt213872-bib-0081] Volpatti, L.R. , and Yetisen, A.K. (2014) Commercialization of microfluidic devices. Trends Biotechnol 32: 347–350. 24954000 10.1016/j.tibtech.2014.04.010

[mbt213872-bib-0082] Ward, J.H. (1963) Hierarchical grouping to optimize an objective function. J Am Stat Assoc 58: 236–244.

[mbt213872-bib-0083] Wasserman, H.H. , Keggi, J.J. , and McKeon, J.E. (1961) Serratamolide, a metabolic product of Serratia. J Am Chem Soc 83: 4107–4108.

[mbt213872-bib-0084] Wiegand, S. , Jogler, M. , Boedeker, C. , Pinto, D. , Vollmers, J. , Rivas‐Marín, E. , *et* *al*. (2019) Cultivation and functional characterization of 79 planctomycetes uncovers their unique biology. Nat Microbiol 5: 126–140. 31740763 10.1038/s41564-019-0588-1 PMC7286433

[mbt213872-bib-0085] Yang, J.Y. , Sanchez, L.M. , Rath, C.M. , Liu, X. , Boudreau, P.D. , Bruns, N. , *et* *al*. (2013) Molecular networking as a dereplication strategy. J Nat Prod 76: 1686–1699. 24025162 10.1021/np400413s PMC3936340

[mbt213872-bib-0086] Zengler, K. , Toledo, G. , Rappe, M. , Elkins, J. , Mathur, E.J. , Short, J.M. , and Keller, M. (2002) Cultivating the uncultured. Proc Natl Acad Sci USA 99: 15681–15686. 12438682 10.1073/pnas.252630999 PMC137776

[mbt213872-bib-0087] Zinchenko, A. , Devenish, S.R. , Kintses, B. , Colin, P.Y. , Fischlechner, M. , and Hollfelder, F. (2014) One in a million: flow cytometric sorting of single cell‐lysate assays in monodisperse picolitre double emulsion droplets for directed evolution. Anal Chem 86: 2526–2533. 24517505 10.1021/ac403585p PMC3952496

[mbt213872-bib-0088] Zizika, Z. (1998) Biological effects of macrotetrolide antibiotics and nonactic acids. Folia Microbiol 43: 7–14. 9569622 10.1007/BF02815533

