# Supplementary material for: Combination of high‐throughput microfluidics and FACS technologies to leverage the numbers game in natural product discovery
Source: Microb Biotechnol. 2021 Jun 24;15(2):415–30. doi: 10.1111/1751-7915.13872 (PMC8867984; doi:10.1111/1751-7915.13872)
Supplement: Supplementary file 1 — Fig. S1. Poisson distribution of λ0.1 and λ10 showing the probability of the amount of cells encapsulated per droplet. Fig. S2. Work scheme of this study showing the microfluidics workflow, the subsequent cultivation, screening and dereplication Fig. S3. Live/dead staining using the LIVE/DEAD BacLight Bacterial Viability and Counting Kit (L7007, Invitrogen). Manufacturer’s protocol was applied on the cells retrieved by nycodenz densitiy gradient centrifugation (A: SYTO 9, B: propidium iodide, C: merged) directly after bacterial isolation. Exemplary pictures are shown, ten independent stains were done and considered for the calculation. Live:dead ratio was estimated resulting in ~ 70:30 ± 6.7%. Fig. S4. Phylogenetic classification of FHG110511 within the phylum Acidobacteria clustering into subgroup 1. The tree is based on a ClustalW alignment of available 16S rRNA gene sequences from the ref_seq database between positions 113 and 1357 [based on Escherichia coli 16S rRNA gene numbering (Brosius et al., 1978)] from the most similar sequences to the isolated strains, and also includes representatives of Acidobacteria subgroups 1, 3, 4, 6, 7, 8, and 10. The tree was calculated using Mega v7.0.26 with the maximum‐likelihood method and GTR‐Gamma model. Circles on the tree branches indicate values of 1000 bootstrap replicates with a bootstrap support of more than 50%. Subgroup affiliations are indicated by colors. The new isolate is indicated by a black arrow. The tree is drawn to scale, with branch lengths measured in the number of substitutions per site. Fig. S5. (A) Assay read‐out of μ‐fractionation plates of strain FHG110488 against M. smegmatis ATCC 607. Fractions are numbered and those causing at least 70% rel. growth inhibition were considered “active” and marked red. Column 1: medium control; Column 2+3: antibiotic standard (isoniazid); Column 4: growth control. Area AH05‐AH24 top: 5 μl injection volume; Area AH05‐AH24 bottom: 10 μl injection; Crude: crude extract [file MBT2-15-415-s001.docx]

**Supplementary material**

**Combination of high-throughput Microfluidics- and FACS technologies to leverage the numbers game in natural product discovery**

Markus Oberpaul^1,λ^, Stephan Brinkmann^1,λ^, Michael Marner^1^, Sanja Mihajlovic^1^, Benedikt Leis^1^, Maria A. Patras^1^, Christoph Hartwig^1^, Andreas Vilcinskas^1,2^, Peter E. Hammann^3^, Till F. Schäberle^1,2,4*^, Marius Spohn^1,**^ and Jens Glaeser^3,***^

^1^ Fraunhofer Institute for Molecular Biology and Applied Ecology (IME), Branch for Bioresources, 35392 Giessen, Germany

^2^ Institute for Insect Biotechnology, Justus-Liebig-University-Giessen, 35392 Giessen, Germany

^3^ Evotec International GmbH, 37079 Göttingen, Germany

^4^ German Center for Infection Research (DZIF), partner site Giessen-Marburg-Langen, 35392 Giessen, Germany

Correspondence: *Till F. Schäberle [Till.F.Schaeberle@agrar.uni-giessen.de](mailto:Till.F.Schaeberle@agrar.uni-giessen.de); **Marius Spohn [marius.spohn@ime.fraunhofer.de](mailto:marius.spohn@ime.fraunhofer.de); ***Jens Glaeser [Jens.Glaeser@evotec.com](mailto:Jens.Glaeser@evotec.com);

^λ^ Authors contributed equally

**Running title:** Microfluidics-FACS bioprospecting platform

**Keywords:** Microfluidics, FACS, high-throughput cultivation, underexplored bacterial phyla, natural product discovery, antimycobacterial, crop disease management, Acidobacteria

**Supplementary Fig. S1.** Poisson distribution of λ0.1 and λ10 showing the probability of the amount of cells encapsulated per droplet.

**Supplementary Fig. S2.** Work scheme of this study showing the microfluidics workflow, the subsequent cultivation, screening and dereplication

**Supplementary Fig. S3.** Live/dead staining using the LIVE/DEAD BacLight Bacterial Viability and Counting Kit (L7007, Invitrogen).

**Supplementary Tab. S1.** Overview of the cultured genera separated by sample and media.

**Supplementary Fig. S4.** Phylogenetic classification of FHG110511 within the phylum Acidobacteria.

**Supplementary Tab. S2.** Cosine similarity table – Data for Fig. affiliation

**Supplementary Fig. S5.** Assay read-out of μ-fractionation plates of strain FHG110488 against *M. smegmatis* ATCC 607.

**Supplementary Fig. S6.** MS2-network of “active” extract of FHG110488 against *Septoria tritici* MUCL45407 focusing on the cluster representing all seven detected serratamolide derivatives and their literature known structures.

**Supplementary Fig. S7.** Assay read-out of fractions 104+105, 107-113 and 116 of μ-fractionation plate of strain FHG110502 against *Mycobacterium smegmatis* ATCC 607.

**Supplementary Fig. S8.** Assay read-out of fractions 92+93 and 130-135 of μ-fractionation plate of strain FHG110502 against *Mycobacterium smegmatis* ATCC 607.

**Supplementary Fig. S9.** Assay read-out of μ-fractionation plate of strain FHG110508 against *Staphylococcus aureus* ATCC 25923.

**Supplementary Fig. S10.** MS2-network of “active” extract of FHG110508 against *Staphylococcus aureus* ATCC 25923 with focus on the cluster representing all five detected macrotetrolide derivatives and their adduct ions.


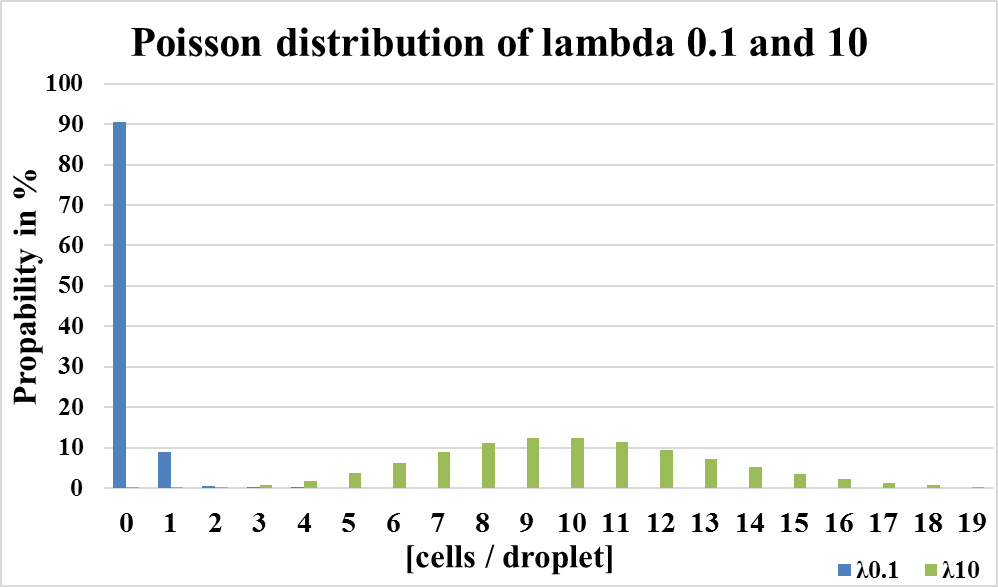


**Supplementary Fig. S1.** Poisson distribution of λ0.1 and λ10 showing the probability of the amount of cells encapsulated per droplet.


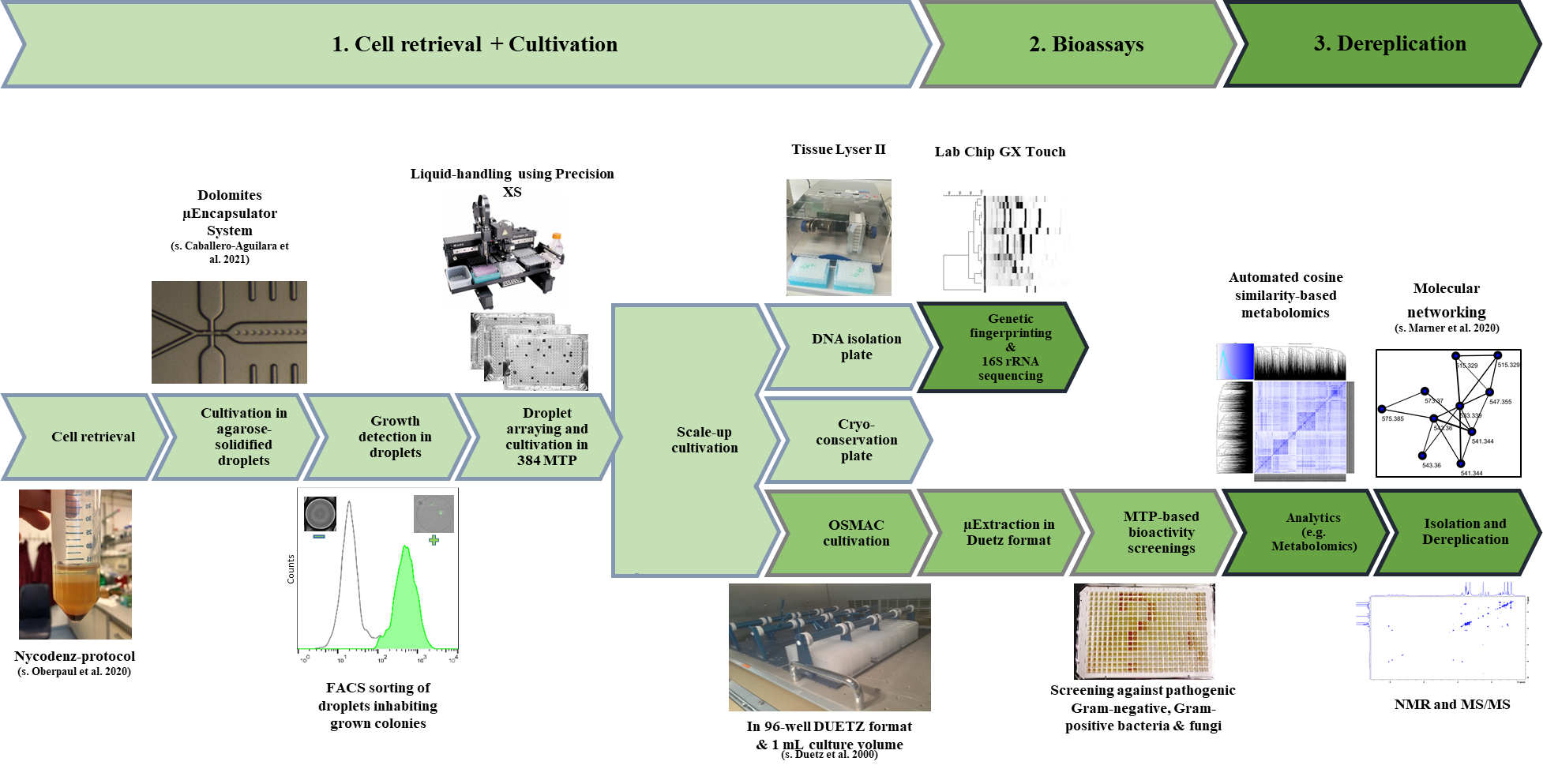
 (Marner *et al.* 2020; Oberpaul *et al.* 2020; Caballero-Aguilara *et al.* 2021; Duetz *et al.* 2000)

**Supplementary Fig. S2.** Work scheme of this study showing the microfluidics workflow, the subsequent cultivation, screening and dereplication.


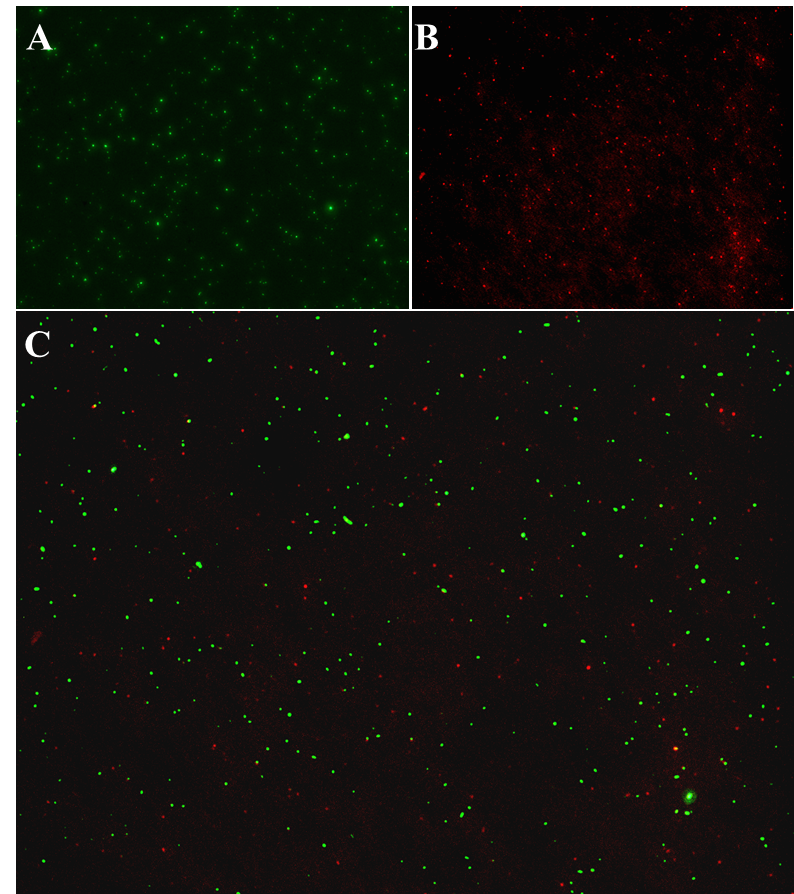


**Supplementary Fig. S3.** Live/dead staining using the LIVE/DEAD BacLight Bacterial Viability and Counting Kit (L7007, Invitrogen). Manufacturer’s protocol was applied on the cells retrieved by nycodenz densitiy gradient centrifugation (**A**: SYTO 9, **B**: propidium iodide, **C**: merged) directly after bacterial isolation. Exemplary pictures are shown, ten independent stains were done and considered for the calculation. Live:dead ratio was estimated resulting in ~70:30 ± 6.7%.

**Tab. S1.** Overview of the cultured genera in mISEM^2^ pH 7.2, pH 5.5 and VL55-xyl pH 5.5. 16*S* rRNA gene sequencing was applied using the primer 1492R on mechanically disrupted culture broth grown for 7 days

| **Phylum** | **Class** | **Genus** | **No. of identified sequences** | **Collection date** | **Sampling site** | **Sampling coordinates** | **Local environment** | **Isolation medium** |
| --- | --- | --- | --- | --- | --- | --- | --- | --- |
| Acidobacteria | Acidobacteriia | *Edaphobacter* | 2 | 29.01.2019 | Bergwerkswald Gießen, Germany | 50.564032 N, 8.672555 E | forrest soil | VL55-Xylan |
| Actinobacteria | Actinobacteria | *Mycobacterium* | 4 | 29.01.2019 | Bergwerkswald Gießen, Germany | 50.564032 N, 8.672555 E | forrest soil | VL55-Xylan |
| Actinobacteria | Actinobacteria | *Cellulomonas* | 1 | 29.01.2019 | Bergwerkswald Gießen, Germany | 50.564032 N, 8.672555 E | forrest soil | VL55-Xylan |
| Actinobacteria | Actinobacteria | *Nocardioides* | 7 | 29.01.2019 | Bergwerkswald Gießen, Germany | 50.564032 N, 8.672555 E | forrest soil | VL55-Xylan |
| Actinobacteria | Actinobacteria | *Rothia* | 1 | 29.01.2019 | Bergwerkswald Gießen, Germany | 50.564032 N, 8.672555 E | forrest soil | VL55-Xylan |
| Actinobacteria | Actinobacteria | *Streptomyces* | 2 | 29.01.2019 | Bergwerkswald Gießen, Germany | 50.564032 N, 8.672555 E | forrest soil | VL55-Xylan |
| Actinobacteria | Actinobacteria | *Mycolicibacterium* | 2 | 29.01.2019 | Bergwerkswald Gießen, Germany | 50.564032 N, 8.672555 E | forrest soil | VL55-Xylan |
| Bacteroidetes | Sphingobacteriia | *Mucilaginibacter* | 5 | 29.01.2019 | Bergwerkswald Gießen, Germany | 50.564032 N, 8.672555 E | forrest soil | VL55-Xylan |
| Firmicutes | Clostridia | *Clostridium* | 1 | 29.01.2019 | Bergwerkswald Gießen, Germany | 50.564032 N, 8.672555 E | forrest soil | VL55-Xylan |
| Proteobacteria | Alphaproteobacteria | *Ancylobacter* | 1 | 29.01.2019 | Bergwerkswald Gießen, Germany | 50.564032 N, 8.672555 E | forrest soil | VL55-Xylan |
| Proteobacteria | Alphaproteobacteria | *Bradyrhizobium* | 3 | 29.01.2019 | Bergwerkswald Gießen, Germany | 50.564032 N, 8.672555 E | forrest soil | VL55-Xylan |
| Proteobacteria | Alphaproteobacteria | *Inquilinus* | 2 | 29.01.2019 | Bergwerkswald Gießen, Germany | 50.564032 N, 8.672555 E | forrest soil | VL55-Xylan |
| Proteobacteria | Alphaproteobacteria | *Kaistia* | 1 | 29.01.2019 | Bergwerkswald Gießen, Germany | 50.564032 N, 8.672555 E | forrest soil | VL55-Xylan |
| Proteobacteria | Alphaproteobacteria | *Labrys* | 9 | 29.01.2019 | Bergwerkswald Gießen, Germany | 50.564032 N, 8.672555 E | forrest soil | VL55-Xylan |
| Proteobacteria | Alphaproteobacteria | *Mesorhizobium* | 30 | 29.01.2019 | Bergwerkswald Gießen, Germany | 50.564032 N, 8.672555 E | forrest soil | VL55-Xylan |
| Proteobacteria | Alphaproteobacteria | *Phyllobacterium* | 98 | 29.01.2019 | Bergwerkswald Gießen, Germany | 50.564032 N, 8.672555 E | forrest soil | VL55-Xylan |
| Proteobacteria | Alphaproteobacteria | *Rhizobium* | 42 | 29.01.2019 | Bergwerkswald Gießen, Germany | 50.564032 N, 8.672555 E | forrest soil | VL55-Xylan |
| Proteobacteria | Alphaproteobacteria | *Sphingomonas* | 3 | 29.01.2019 | Bergwerkswald Gießen, Germany | 50.564032 N, 8.672555 E | forrest soil | VL55-Xylan |
| Proteobacteria | Betaproteobacteria | *Burkholderia* | 6 | 29.01.2019 | Bergwerkswald Gießen, Germany | 50.564032 N, 8.672555 E | forrest soil | VL55-Xylan |
| Proteobacteria | Betaproteobacteria | *Variovorax* | 1 | 29.01.2019 | Bergwerkswald Gießen, Germany | 50.564032 N, 8.672555 E | forrest soil | VL55-Xylan |
| Proteobacteria | Betaproteobacteria | *Caballeronia* | 1 | 29.01.2019 | Bergwerkswald Gießen, Germany | 50.564032 N, 8.672555 E | forrest soil | VL55-Xylan |
| Proteobacteria | Gammaproteobacteria | *Moraxella* | 1 | 29.01.2019 | Bergwerkswald Gießen, Germany | 50.564032 N, 8.672555 E | forrest soil | VL55-Xylan |
| Proteobacteria | Gammaproteobacteria | *Luteibacter* | 1 | 29.01.2019 | Bergwerkswald Gießen, Germany | 50.564032 N, 8.672555 E | forrest soil | VL55-Xylan |
| Proteobacteria | Gammaproteobacteria | *Dyella* | 1 | 29.01.2019 | Bergwerkswald Gießen, Germany | 50.564032 N, 8.672555 E | forrest soil | VL55-Xylan |
|  |  | No Relative /no sequence data | 117 | 29.01.2019 | Bergwerkswald Gießen, Germany | 50.564032 N, 8.672555 E | forrest soil | VL55-Xylan |
|  |  |  |  |  |  |  |  |  |
| Acidobacteria | Acidobacteriia | *Edaphobacter* | 6 | 29.01.2019 | Bergwerkswald Gießen, Germany | 50.564032 N, 8.672555 E | forrest soil | mISEM^2^  pH 5.5 |
| Actinobacteria | Actinobacteria | *Agromyces* | 7 | 29.01.2019 | Bergwerkswald Gießen, Germany | 50.564032 N, 8.672555 E | forrest soil | mISEM^2^  pH 5.5 |
| Actinobacteria | Actinobacteria | *Angustibacter* | 2 | 29.01.2019 | Bergwerkswald Gießen, Germany | 50.564032 N, 8.672555 E | forrest soil | mISEM^2^  pH 5.5 |
| Actinobacteria | Actinobacteria | *Cellulomonas* | 2 | 29.01.2019 | Bergwerkswald Gießen, Germany | 50.564032 N, 8.672555 E | forrest soil | mISEM^2^  pH 5.5 |
| Actinobacteria | Actinobacteria | *Leifsonia* | 1 | 29.01.2019 | Bergwerkswald Gießen, Germany | 50.564032 N, 8.672555 E | forrest soil | mISEM^2^  pH 5.5 |
| Actinobacteria | Actinobacteria | *Mycobacterium* | 4 | 29.01.2019 | Bergwerkswald Gießen, Germany | 50.564032 N, 8.672555 E | forrest soil | mISEM^2^  pH 5.5 |
| Actinobacteria | Actinobacteria | *Mycolicibacterium* | 2 | 29.01.2019 | Bergwerkswald Gießen, Germany | 50.564032 N, 8.672555 E | forrest soil | mISEM^2^  pH 5.5 |
| Actinobacteria | Actinobacteria | *Nocardioides* | 2 | 29.01.2019 | Bergwerkswald Gießen, Germany | 50.564032 N, 8.672555 E | forrest soil | mISEM^2^  pH 5.5 |
| Actinobacteria | Actinobacteria | *Rhodococcus* | 1 | 29.01.2019 | Bergwerkswald Gießen, Germany | 50.564032 N, 8.672555 E | forrest soil | mISEM^2^  pH 5.5 |
| Bacteroidetes | Sphingobacteriia | *Mucilaginibacter* | 3 | 29.01.2019 | Bergwerkswald Gießen, Germany | 50.564032 N, 8.672555 E | forrest soil | mISEM^2^  pH 5.5 |
| Proteobacteria | Alphaproteobacteria | *Ancylobacter* | 9 | 29.01.2019 | Bergwerkswald Gießen, Germany | 50.564032 N, 8.672555 E | forrest soil | mISEM^2^  pH 5.5 |
| Proteobacteria | Alphaproteobacteria | *Bosea* | 1 | 29.01.2019 | Bergwerkswald Gießen, Germany | 50.564032 N, 8.672555 E | forrest soil | mISEM^2^  pH 5.5 |
| Proteobacteria | Betaproteobacteria | *Burkholderia* | 1 | 29.01.2019 | Bergwerkswald Gießen, Germany | 50.564032 N, 8.672555 E | forrest soil | mISEM^2^  pH 5.5 |
| Proteobacteria | Gammaproteobacteria | *Buttiauxella* | 1 | 29.01.2019 | Bergwerkswald Gießen, Germany | 50.564032 N, 8.672555 E | forrest soil | mISEM^2^  pH 5.5 |
| Proteobacteria | Betaproteobacteria | *Caballeronia* | 2 | 29.01.2019 | Bergwerkswald Gießen, Germany | 50.564032 N, 8.672555 E | forrest soil | mISEM^2^  pH 5.5 |
| Proteobacteria | Betaproteobacteria | *Cupriavidus* | 1 | 29.01.2019 | Bergwerkswald Gießen, Germany | 50.564032 N, 8.672555 E | forrest soil | mISEM^2^  pH 5.5 |
| Proteobacteria | Gammaproteobacteria | *Dyella* | 1 | 29.01.2019 | Bergwerkswald Gießen, Germany | 50.564032 N, 8.672555 E | forrest soil | mISEM^2^  pH 5.5 |
| Proteobacteria | Alphaproteobacteria | *Inquilinus* | 1 | 29.01.2019 | Bergwerkswald Gießen, Germany | 50.564032 N, 8.672555 E | forrest soil | mISEM^2^  pH 5.5 |
| Proteobacteria | Gammaproteobacteria | *Luteibacter* | 5 | 29.01.2019 | Bergwerkswald Gießen, Germany | 50.564032 N, 8.672555 E | forrest soil | mISEM^2^  pH 5.5 |
| Proteobacteria | Alphaproteobacteria | *Mesorhizobium* | 11 | 29.01.2019 | Bergwerkswald Gießen, Germany | 50.564032 N, 8.672555 E | forrest soil | mISEM^2^  pH 5.5 |
| Proteobacteria | Alphaproteobacteria | *Phyllobacterium* | 25 | 29.01.2019 | Bergwerkswald Gießen, Germany | 50.564032 N, 8.672555 E | forrest soil | mISEM^2^  pH 5.5 |
| Proteobacteria | Alphaproteobacteria | *Rhizobium* | 6 | 29.01.2019 | Bergwerkswald Gießen, Germany | 50.564032 N, 8.672555 E | forrest soil | mISEM^2^  pH 5.5 |
| Proteobacteria | Alphaproteobacteria | *Sphingomonas* | 9 | 29.01.2019 | Bergwerkswald Gießen, Germany | 50.564032 N, 8.672555 E | forrest soil | mISEM^2^  pH 5.5 |
| Proteobacteria | Betaproteobacteria | *Variovorax* | 3 | 29.01.2019 | Bergwerkswald Gießen, Germany | 50.564032 N, 8.672555 E | forrest soil | mISEM^2^  pH 5.5 |
|  |  | No Relative /no sequence data | 20 | 29.01.2019 | Bergwerkswald Gießen, Germany | 50.564032 N, 8.672555 E | forrest soil | mISEM^2^  pH 5.5 |
|  |  |  |  |  |  |  |  |  |
| Proteobacteria | Betaproteobacteria | *Achromobacter* | 1 | 29.01.2019 | Bergwerkswald Gießen, Germany | 50.564032 N, 8.672555 E | forrest soil | mISEM^2^  pH 7.2 |
| Actinobacteria | Actinobacteria | *Agromyces* | 151 | 29.01.2019 | Bergwerkswald Gießen, Germany | 50.564032 N, 8.672555 E | forrest soil | mISEM^2^  pH 7.2 |
| Actinobacteria | Actinobacteria | *Arthrobacter* | 1 | 29.01.2019 | Bergwerkswald Gießen, Germany | 50.564032 N, 8.672555 E | forrest soil | mISEM^2^  pH 7.2 |
| Actinobacteria | Actinobacteria | *Cellulomonas* | 6 | 29.01.2019 | Bergwerkswald Gießen, Germany | 50.564032 N, 8.672555 E | forrest soil | mISEM^2^  pH 7.2 |
| Actinobacteria | Actinobacteria | *Glaciibacter* | 19 | 29.01.2019 | Bergwerkswald Gießen, Germany | 50.564032 N, 8.672555 E | forrest soil | mISEM^2^  pH 7.2 |
| Actinobacteria | Actinobacteria | *Isoptericola* | 1 | 29.01.2019 | Bergwerkswald Gießen, Germany | 50.564032 N, 8.672555 E | forrest soil | mISEM^2^  pH 7.2 |
| Actinobacteria | Actinobacteria | *Leifsonia* | 2 | 29.01.2019 | Bergwerkswald Gießen, Germany | 50.564032 N, 8.672555 E | forrest soil | mISEM^2^  pH 7.2 |
| Actinobacteria | Actinobacteria | *Microbacterium* | 12 | 29.01.2019 | Bergwerkswald Gießen, Germany | 50.564032 N, 8.672555 E | forrest soil | mISEM^2^  pH 7.2 |
| Actinobacteria | Actinobacteria | *Mycetocola* | 2 | 29.01.2019 | Bergwerkswald Gießen, Germany | 50.564032 N, 8.672555 E | forrest soil | mISEM^2^  pH 7.2 |
| Actinobacteria | Actinobacteria | *Mycobacterium* | 6 | 29.01.2019 | Bergwerkswald Gießen, Germany | 50.564032 N, 8.672555 E | forrest soil | mISEM^2^  pH 7.2 |
| Actinobacteria | Actinobacteria | *Nakamurella* | 1 | 29.01.2019 | Bergwerkswald Gießen, Germany | 50.564032 N, 8.672555 E | forrest soil | mISEM^2^  pH 7.2 |
| Actinobacteria | Actinobacteria | *Nocardioides* | 3 | 29.01.2019 | Bergwerkswald Gießen, Germany | 50.564032 N, 8.672555 E | forrest soil | mISEM^2^  pH 7.2 |
| Actinobacteria | Actinobacteria | *Plantibacter* | 2 | 29.01.2019 | Bergwerkswald Gießen, Germany | 50.564032 N, 8.672555 E | forrest soil | mISEM^2^  pH 7.2 |
| Actinobacteria | Actinobacteria | *Promicromonospora* | 2 | 29.01.2019 | Bergwerkswald Gießen, Germany | 50.564032 N, 8.672555 E | forrest soil | mISEM^2^  pH 7.2 |
| Actinobacteria | Actinobacteria | *Rhodococcus* | 2 | 29.01.2019 | Bergwerkswald Gießen, Germany | 50.564032 N, 8.672555 E | forrest soil | mISEM^2^  pH 7.2 |
| Actinobacteria | Actinobacteria | *Streptomyces* | 2 | 29.01.2019 | Bergwerkswald Gießen, Germany | 50.564032 N, 8.672555 E | forrest soil | mISEM^2^  pH 7.2 |
| Actinobacteria | Actinobacteria | *Subtercola* | 1 | 29.01.2019 | Bergwerkswald Gießen, Germany | 50.564032 N, 8.672555 E | forrest soil | mISEM^2^  pH 7.2 |
| Actinobacteria | Actinobacteria | *Williamsia* | 2 | 29.01.2019 | Bergwerkswald Gießen, Germany | 50.564032 N, 8.672555 E | forrest soil | mISEM^2^  pH 7.2 |
| Bacteroidetes | Flavobacterium | *Flavobacterium* | 1 | 29.01.2019 | Bergwerkswald Gießen, Germany | 50.564032 N, 8.672555 E | forrest soil | mISEM^2^  pH 7.2 |
| Bacteroidetes | Sphingobacteriia | *Pedobacter* | 2 | 29.01.2019 | Bergwerkswald Gießen, Germany | 50.564032 N, 8.672555 E | forrest soil | mISEM^2^  pH 7.2 |
| Bacteroidetes | Chitinophaga | *Pseudoflavitalea* | 1 | 29.01.2019 | Bergwerkswald Gießen, Germany | 50.564032 N, 8.672555 E | forrest soil | mISEM^2^  pH 7.2 |
| Firmicutes | Bacilli | *Staphylococcus* | 1 | 29.01.2019 | Bergwerkswald Gießen, Germany | 50.564032 N, 8.672555 E | forrest soil | mISEM^2^  pH 7.2 |
| Proteobacteria | Alphaproteobacteria | *Ancylobacter* | 112 | 29.01.2019 | Bergwerkswald Gießen, Germany | 50.564032 N, 8.672555 E | forrest soil | mISEM^2^  pH 7.2 |
| Proteobacteria | Alphaproteobacteria | *Nitratireductor* | 43 | 29.01.2019 | Bergwerkswald Gießen, Germany | 50.564032 N, 8.672555 E | forrest soil | mISEM^2^  pH 7.2 |
| Proteobacteria | Alphaproteobacteria | *Bosea* | 1 | 29.01.2019 | Bergwerkswald Gießen, Germany | 50.564032 N, 8.672555 E | forrest soil | mISEM^2^  pH 7.2 |
| Proteobacteria | Alphaproteobacteria | *Bradyrhizobium* | 8 | 29.01.2019 | Bergwerkswald Gießen, Germany | 50.564032 N, 8.672555 E | forrest soil | mISEM^2^  pH 7.2 |
| Proteobacteria | Betaproteobacteria | *Burkholderia* | 7 | 29.01.2019 | Bergwerkswald Gießen, Germany | 50.564032 N, 8.672555 E | forrest soil | mISEM^2^  pH 7.2 |
| Proteobacteria | Betaproteobacteria | *Cupriavidus* | 1 | 29.01.2019 | Bergwerkswald Gießen, Germany | 50.564032 N, 8.672555 E | forrest soil | mISEM^2^  pH 7.2 |
| Proteobacteria | Alphaproteobacteria | *Hyphomicrobium* | 2 | 29.01.2019 | Bergwerkswald Gießen, Germany | 50.564032 N, 8.672555 E | forrest soil | mISEM^2^  pH 7.2 |
| Proteobacteria | Alphaproteobacteria | *Inquilinus* | 1 | 29.01.2019 | Bergwerkswald Gießen, Germany | 50.564032 N, 8.672555 E | forrest soil | mISEM^2^  pH 7.2 |
| Proteobacteria | Alphaproteobacteria | *Kaistia* | 1 | 29.01.2019 | Bergwerkswald Gießen, Germany | 50.564032 N, 8.672555 E | forrest soil | mISEM^2^  pH 7.2 |
| Proteobacteria | Gammaproteobacteria | *Luteibacter* | 10 | 29.01.2019 | Bergwerkswald Gießen, Germany | 50.564032 N, 8.672555 E | forrest soil | mISEM^2^  pH 7.2 |
| Proteobacteria | Gammaproteobacteria | *Lysobacter* | 2 | 29.01.2019 | Bergwerkswald Gießen, Germany | 50.564032 N, 8.672555 E | forrest soil | mISEM^2^  pH 7.2 |
| Proteobacteria | Alphaproteobacteria | *Mesorhizobium* | 3 | 29.01.2019 | Bergwerkswald Gießen, Germany | 50.564032 N, 8.672555 E | forrest soil | mISEM^2^  pH 7.2 |
| Proteobacteria | Betaproteobacteria | *Paraburkholderia* | 1 | 29.01.2019 | Bergwerkswald Gießen, Germany | 50.564032 N, 8.672555 E | forrest soil | mISEM^2^  pH 7.2 |
| Proteobacteria | Alphaproteobacteria | *Phyllobacterium* | 2 | 29.01.2019 | Bergwerkswald Gießen, Germany | 50.564032 N, 8.672555 E | forrest soil | mISEM^2^  pH 7.2 |
| Proteobacteria | Betaproteobacteria | *Polaromonas* | 1 | 29.01.2019 | Bergwerkswald Gießen, Germany | 50.564032 N, 8.672555 E | forrest soil | mISEM^2^  pH 7.2 |
| Proteobacteria | Gammaproteobacteria | *Pseudomonas* | 10 | 29.01.2019 | Bergwerkswald Gießen, Germany | 50.564032 N, 8.672555 E | forrest soil | mISEM^2^  pH 7.2 |
| Proteobacteria | Gammaproteobacteria | *Pseudoxanthomonas* | 1 | 29.01.2019 | Bergwerkswald Gießen, Germany | 50.564032 N, 8.672555 E | forrest soil | mISEM^2^  pH 7.2 |
| Proteobacteria | Alphaproteobacteria | *Reyranella* | 1 | 29.01.2019 | Bergwerkswald Gießen, Germany | 50.564032 N, 8.672555 E | forrest soil | mISEM^2^  pH 7.2 |
| Proteobacteria | Alphaproteobacteria | *Rhizobium* | 9 | 29.01.2019 | Bergwerkswald Gießen, Germany | 50.564032 N, 8.672555 E | forrest soil | mISEM^2^  pH 7.2 |
| Proteobacteria | Alphaproteobacteria | *Rhodopseudomonas* | 1 | 29.01.2019 | Bergwerkswald Gießen, Germany | 50.564032 N, 8.672555 E | forrest soil | mISEM^2^  pH 7.2 |
| Proteobacteria | Alphaproteobacteria | *Sinorhizobium/Ensifer* | 2 | 29.01.2019 | Bergwerkswald Gießen, Germany | 50.564032 N, 8.672555 E | forrest soil | mISEM^2^  pH 7.2 |
| Proteobacteria | Alphaproteobacteria | *Sphingomonas* | 1 | 29.01.2019 | Bergwerkswald Gießen, Germany | 50.564032 N, 8.672555 E | forrest soil | mISEM^2^  pH 7.2 |
| Proteobacteria | Betaproteobacteria | *Variovorax* | 18 | 29.01.2019 | Bergwerkswald Gießen, Germany | 50.564032 N, 8.672555 E | forrest soil | mISEM^2^  pH 7.2 |
| Proteobacteria | Gammaproteobacteria | *Xanthomonas* | 3 | 29.01.2019 | Bergwerkswald Gießen, Germany | 50.564032 N, 8.672555 E | forrest soil | mISEM^2^  pH 7.2 |
|  |  | No Relative/no sequence data total | 141 | 29.01.2019 | Bergwerkswald Gießen, Germany | 50.564032 N, 8.672555 E | forrest soil | mISEM^2^  pH 7.2 |
|  |  |  |  |  |  |  |  |  |
|  |  | No Relative /no sequence data total | 278 |  |  |  |  |  |


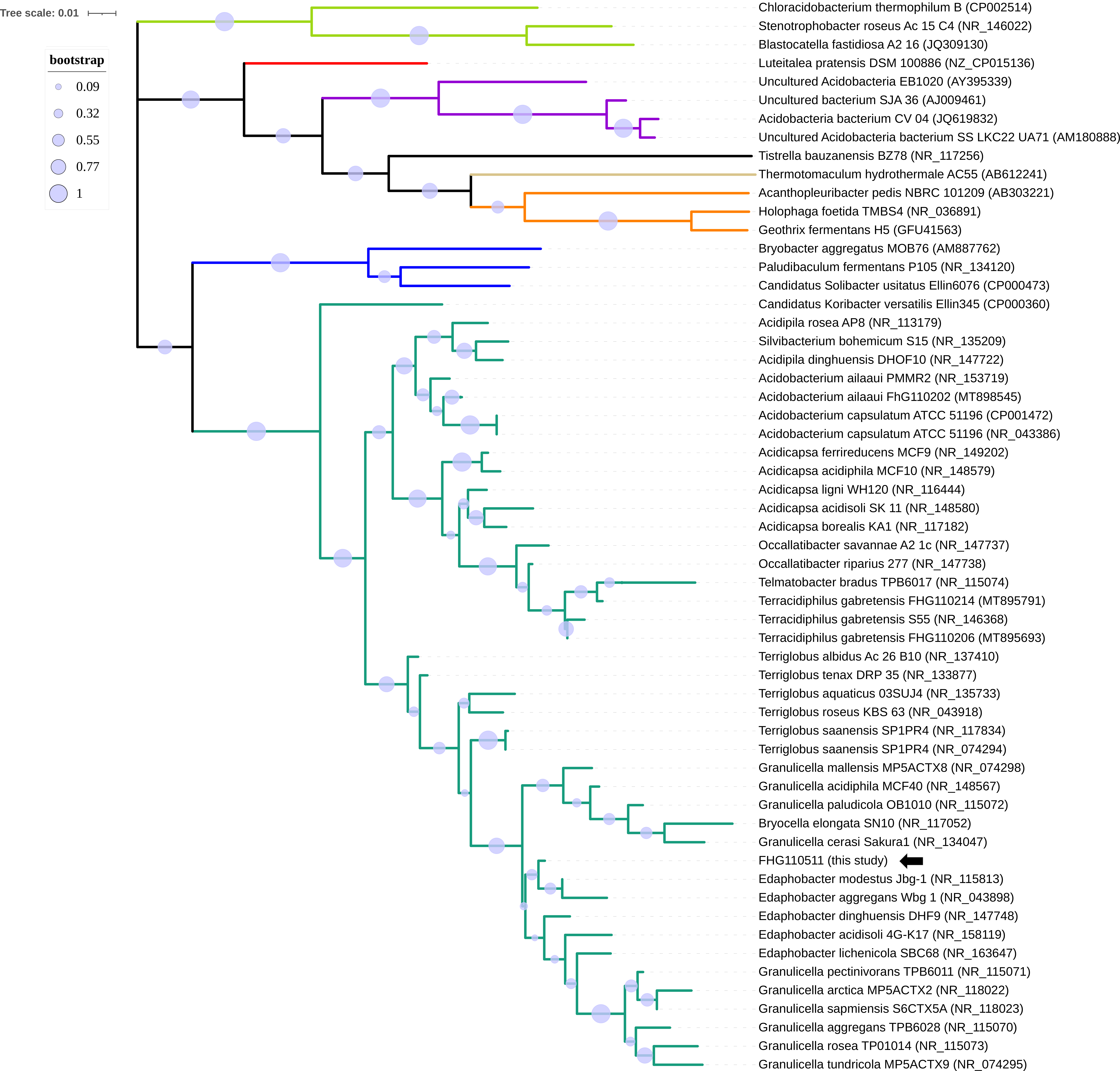


**Supplementary Fig. S4.** Phylogenetic classification of FHG110511 within the phylum Acidobacteria clustering into subgroup 1. The tree is based on a ClustalW alignment of available 16*S* rRNA gene sequences from the ref_seq database between positions 113 and 1,357 [based on Escherichia coli 16S rRNA gene numbering (Brosius et al., 1978)] from the most similar sequences to the isolated strains, and also includes representatives of Acidobacteria subgroups 1, 3, 4, 6, 7, 8, and 10. The tree was calculated using MEGA v7.0.26 with the maximum-likelihood method and GTR-Gamma model. Circles on the tree branches indicate values of 1,000 bootstrap replicates with a bootstrap support of more than 50%. Subgroup affiliations are indicated by colors. The new isolate is indicated by a black arrow. The tree is drawn to scale, with branch lengths measured in the number of substitutions per site.

**Supplementary Tab. S2.** Cosine Similarity table – Data for Fig. affiliation.

| **Data point** | **Pairwise cosine similarity** | **Metabolic group affiliation** |
| --- | --- | --- |
| COD00090_E-07_A1p_P1-E-7_01_49346.d | 0 | 1 |
| COD00090_E-10_A1p_P1-E-10_01_49349.d | 0,996525855 | 1 |
| COD00090_D-04_A1p_P1-D-4_01_49332.d | 0,994506405 | 1 |
| COD00090_D-05_A1p_P1-D-5_01_49333.d | 0,996323071 | 1 |
| COD00090_E-03_A1p_P1-E-3_01_49342.d | 0,989886248 | 1 |
| COD00090_E-06_A1p_P1-E-6_01_49345.d | 0,995331905 | 1 |
| COD00090_E-09_A1p_P1-E-9_01_49348.d | 0,99494112 | 1 |
| COD00090_D-03_A1p_P1-D-3_01_49331.d | 0,988141974 | 1 |
| COD00090_B-04_A1p_P1-B-4_01_49175.d | 0,992537824 | 1 |
| COD00090_B-09_A1p_P1-B-9_01_49187.d | 0,981227556 | 1 |
| COD00090_E-04_A1p_P1-E-4_01_49343.d | 0,983954903 | 1 |
| COD00090_D-07_A1p_P1-D-7_01_49335.d | 0,992699045 | 1 |
| COD00090_D-11_A1p_P1-D-11_01_49339.d | 0,989752564 | 1 |
| COD00090_E-08_A1p_P1-E-8_01_49347.d | 0,989333574 | 1 |
| COD00090_D-08_A1p_P1-D-8_01_49336.d | 0,978173932 | 1 |
| COD00090_C-08_A1p_P1-C-8_01_49310.d | 0,995030321 | 1 |
| COD00090_C-06_A1p_P1-C-6_01_49308.d | 0,990078579 | 1 |
| COD00090_B-06_A1p_P1-B-6_01_49177.d | 0,984810504 | 1 |
| COD00090_C-09_A1p_P1-C-9_01_49327.d | 0,960325517 | 1 |
| COD00090_A-06_A1p_P1-A-6_01_49165.d | 0,956355956 | 1 |
| COD00090_A-07_A1p_P1-A-7_01_49166.d | 0,996660716 | 1 |
| COD00090_A-11_A1p_P1-A-11_01_49170.d | 0,994957861 | 1 |
| COD00090_A-04_A1p_P1-A-4_01_49126.d | 0,98914863 | 1 |
| COD00090_A-09_A1p_P1-A-9_01_49168.d | 0,988868587 | 1 |
| COD00090_A-10_A1p_P1-A-10_01_49169.d | 0,993676537 | 1 |
| COD00090_B-03_A1p_P1-B-3_01_49173.d | 0,98513016 | 1 |
| COD00090_C-03_A1p_P1-C-3_01_49305.d | 0,981021653 | 1 |
| COD00090_A-08_A1p_P1-A-8_01_49167.d | 0,991637074 | 1 |
| COD00090_C-12_A1p_P1-C-12_01_49330.d | 0,991598453 | 1 |
| COD00090_F-11_A1p_P2-F-11_01_49568.d | 0,985669881 | 1 |
| COD00090_E-12_A1p_P1-E-12_01_49351.d | 0,971662968 | 1 |
| COD00090_C-11_A1p_P1-C-11_01_49329.d | 0,973623794 | 1 |
| COD00090_C-10_A1p_P1-C-10_01_49328.d | 0,992987138 | 1 |
| COD00090_B-08_A1p_P1-B-8_01_49186.d | 0,989997964 | 1 |
| COD00090_D-06_A1p_P1-D-6_01_49334.d | 0,986453388 | 1 |
| COD00090_E-05_A1p_P1-E-5_01_49344.d | 0,992392656 | 1 |
| COD00090_F-10_A1p_P2-F-10_01_49567.d | 0,971880085 | 1 |
| COD00090_F-09_A1p_P2-F-9_01_49566.d | 0,995962731 | 1 |
| COD00090_F-08_A1p_P2-F-8_01_49565.d | 0,993330272 | 1 |
| COD00090_C-04_A1p_P1-C-4_01_49306.d | 0,979506743 | 1 |
| COD00090_A-05_A1p_P1-A-5_01_49127.d | 0,972899421 | 1 |
| COD00090_B-10_A1p_P1-B-10_01_49188.d | 0,965508761 | 1 |
| COD00090_B-11_A1p_P1-B-11_01_49189.d | 0,98021242 | 1 |
| COD00090_D-10_A1p_P1-D-10_01_49338.d | 0,970851409 | 1 |
| COD00090_B-12_A1p_P1-B-12_01_49190.d | 0,979346376 | 1 |
| COD00090_F-04_A1p_P1-F-4_01_49353.d | 0,933395646 | 1 |
| COD00090_E-11_A1p_P1-E-11_01_49350.d | 0,970731424 | 1 |
| COD00090_D-12_A1p_P2-D-12_01_64190.d | 0,922166996 | 1 |
| COD00090_D-11_A1p_P2-D-11_01_64189.d | 0,995436642 | 1 |
| COD00090_E-03_A1p_P2-E-3_01_64191.d | 0,987316104 | 1 |
| COD00090_F-05_A1p_P2-F-5_01_49562.d | 0,946636407 | 1 |
| COD00090_F-06_A1p_P2-F-6_01_49563.d | 0,930737067 | 1 |
| COD00090_F-03_A1p_P1-F-3_01_49352.d | 0,828152621 | 2 |
| COD00090_C-05_A1p_P1-C-5_01_49307.d | 0,93614162 | 2 |
| COD00090_D-09_A1p_P1-D-9_01_49337.d | 0,859523476 | 3 |
| COD00090_F-12_A1p_P2-F-12_01_49569.d | 0,846525324 | 4 |
| COD00090_D-12_A1p_P1-D-12_01_49340.d | 0,881502833 | 5 |
| COD00090_G-03_A1p_P2-G-3_01_49571.d | 0,893182206 | 6 |
| COD00090_F-07_A1p_P2-F-7_01_49564.d | 0,661510307 | 7 |
| COD00090_A-12_A1p_P1-A-12_01_49172.d | 0,822655257 | 8 |


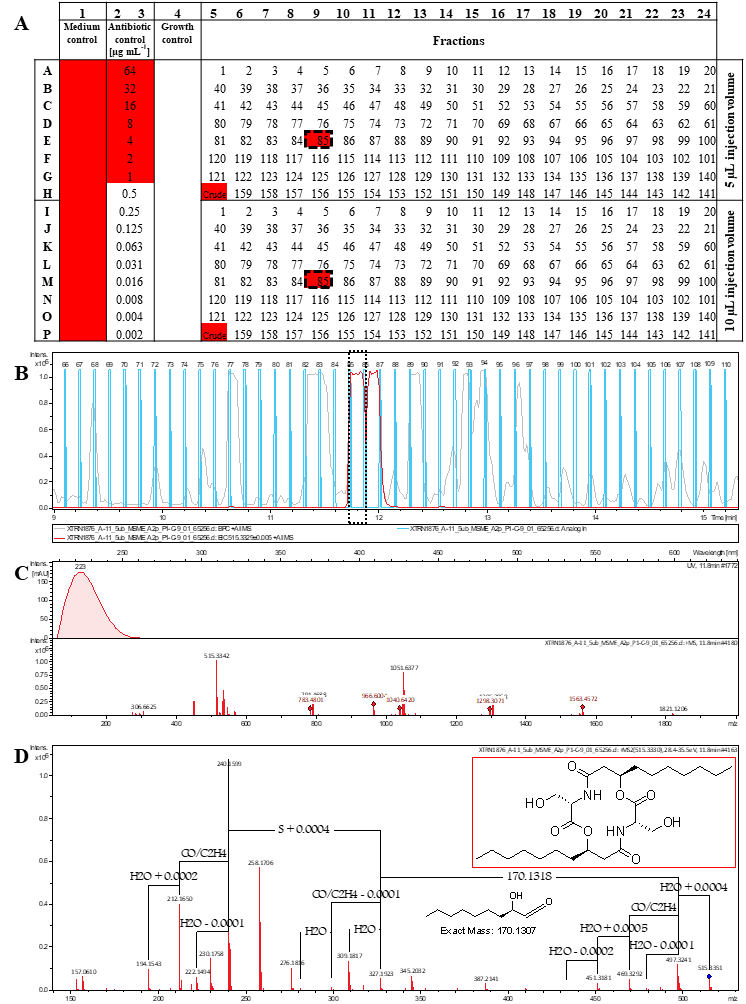


**Supplementary Fig. S5.** (**A**) Assay read-out of μ-fractionation plates of strain FHG110488 against *M. smegmatis* ATCC 607. Fractions are numbered and those causing at least 70% rel. growth inhibition were considered “active” and marked red. Column 1: medium control; Column 2+3: antibiotic standard (isoniazid); Column 4: growth control. Area AH05-AH24 top: 5 μL injection volume; Area AH05-AH24 bottom: 10 μL injection; Crude: crude extract as a control. (**B**) Overlaid Base peak Chromatogram (grey), fraction collector analog signal (light blue bars) and extracted ion chromatogram of *m/z* 515.3329±0.005 [M+H]^+^ (**1**, red) of the 50 fold concentrated extract (in MeOH) with 5 μL injection volume. (**C**) UV and MS spectrum of fractions 84-87. (**D**) MS/MS fragmentation of the precursor ion at *m/z* 515.3330 [M+H]^+^ (dereplicated as Serratamolide A, displayed in red) with manual annotation of the neutral losses.

**
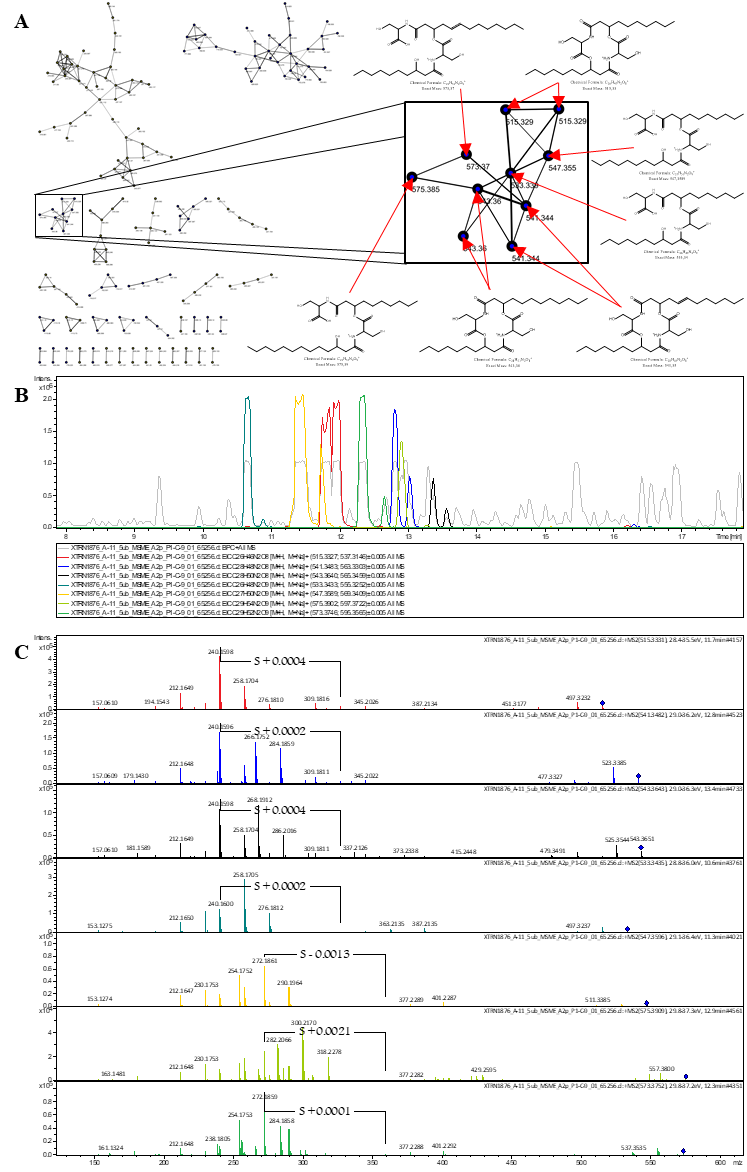
**

**Supplementary Fig. S6.** (**A**) MS2-network of “active” extract of FHG110488 against *Septoria tritici* MUCL45407 focusing on the cluster representing all seven detected serratamolide derivatives and their literature known structures (dots of parent ions found as hits in our internal database or AntiBase are marked in gold). (**B**) Overlaid Base peak Chromatogram (grey) and extracted ion chromatograms of serratamolides **1**-**7** (**1** *m/z* 515.3327 [M+H]^+^, C_26_H_47_N_2_O_8_^+^ (red); **2** *m/z* 541.3483 [M+H]^+^, C_28_H_49_N_2_O_8_^+^ (blue); **3** *m/z* 543.3640 [M+H]^+^, C_28_H_51_N_2_O_8_^+^ (black); **4** *m/z* 533.3433 [M+H]^+^, C_26_H_49_N_2_O_9_^+^ (cyan); **5** *m/z* 547.3598 [M+H]^+^, C_27_H_51_N_2_O_9_^+^ (yellow); **6** *m/z* 575.3902 [M+H]^+^, C_29_H_55_N_2_O_9_^+^ (light green); **7** *m/z* 573.3746 [M+H]^+^, C_29_H_53_N_2_O_9_^+^ (dark green)) of the 50 fold concentrated extract (in MeOH) with 5 μL injection volume. (**C**) MS/MS fragmentation of the precursor ions **1**-**7**.


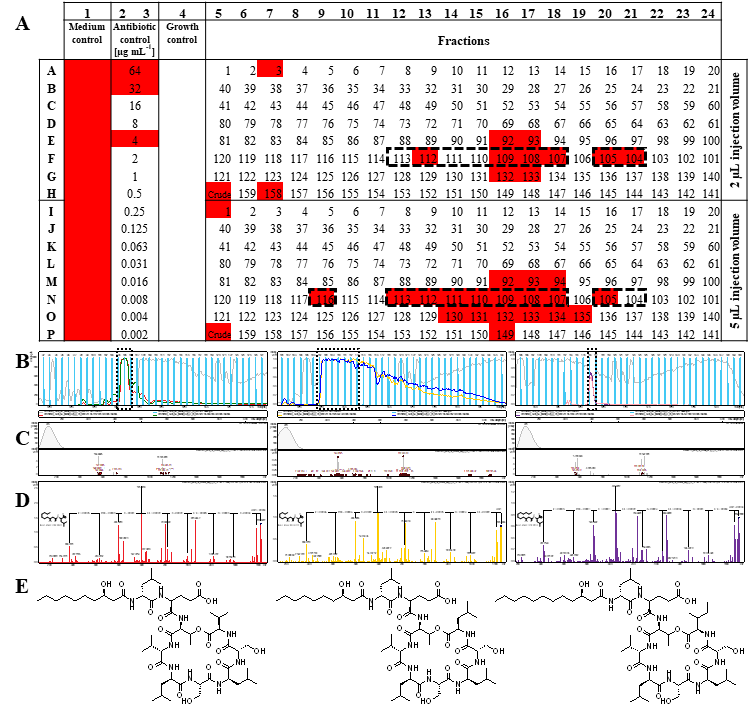


**Supplementary Fig. S7.** (**A**) Assay read-out of μ-fractionation plate of strain FHG110502 against *Mycobacterium smegmatis* ATCC 607. Fractions are numbered and those causing at least 70% rel. growth inhibition were considered “active” and marked red. Column 1: medium control; Column 2+3: antibiotic standard (isoniazid); Column 4: growth control. Area AH05-AH24: 2 μL injection volume; Area IP05-IP24: 5 μL injection; Crude: crude extract as a control. (**B**) Overlaid Base peak Chromatograms (grey), Fraction collector analog signals (light blue bars) and extracted ion chromatogram s of *m/z* 1112.6814±0.005 [M+H]^+^ (**8**, red) with corresponding *m/z* 556.8446±0.005 [M+2H]^2+^ (green), *m/z* 1126.6973±0.005 [M+H]^+^ (**9**, yellow) with corresponding *m/z* 563.8524±0.005 [M+2H]^2+^ (blue), and *m/z* 1154.7288±0.005 [M+H]^+^ (**10**, purple) with corresponding *m/z* 577.8680±0.005 [M+2H]^2+^ (magenta) of the 50 fold concentrated extract (in MeOH) with 5μL injection volume. (**C**) UV and MS spectrum of fractions 105-106 (left), 108-118 (middle) and 116 (right). (**D**) MS/MS fragmentation of the precursor ion at *m/z* 1112.6814 [M+H]^+^, *m/z* 1126.6973 [M+H]^+^, and *m/z* 1154.7288 [M+H]^+^ (dereplicated as massetolide E, massetolide F and massetolide H, respectively), manual annotation of the neutral losses and proposed structures of the fragment ions at *m/z* 284.2229 and *m/z* 312.2533. (**E**) Structures of all three dereplicated compounds **8**-**10**.


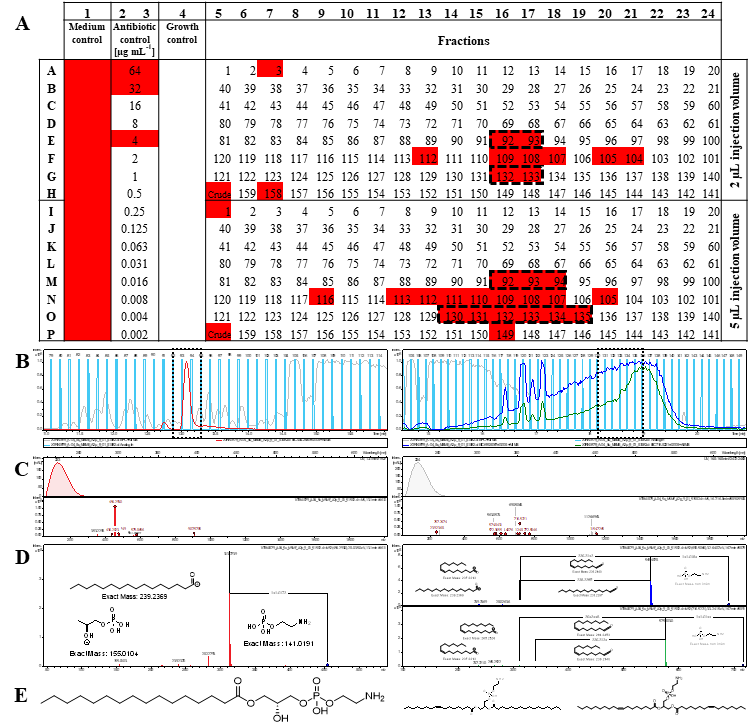


**Supplementary Fig. S8.** (**A**) Assay read-out of μ-fractionation plate of strain FHG110502 against *Mycobacterium smegmatis* ATCC 607. Fractions are numbered and those causing at least 70% rel. growth inhibition were considered “active” and marked red. Column 1: medium control; Column 2+3: antibiotic standard (isoniazid); Column 4: growth control. Area AH05-AH24: 2 μL injection volume; Area IP05-IP24: 5 μL injection; Crude: crude extract as a control. (**B**) Overlaid Base peak Chromatograms (grey), Fraction collector analog signals (light blue bars) and extracted ion chromatogram s of *m/z* 454.2931±0.005 [M+H]^+^ (**11**, red), *m/z* 690.5073±0.005 [M+H]^+^ (**12**, blue) and *m/z* 716.5237±0.005 [M+H]^+^ (**13**, green)of the 50 fold concentrated extract (in MeOH) with 5μL injection volume. (**C**) UV and MS spectrum of fractions 93-94 (left) and 130-136 (right). (**D**) MS/MS fragmentation of the precursor ion at *m/z* 454.2931 [M+H]^+^, *m/z* 690.5073 [M+H]^+^, and *m/z* 716.5237 [M+H]^+^ (dereplicated as lyso-palmitoyl-phosphoethanolamine, palmitoleoyl-palmitoyl-phosphoetanolamine and palmitoleoyl-oleoyl-phosphoetanolamine, respectively), manual annotation of the neutral losses and proposed structures of the fragment ions. (**E**) Putative structures of all three dereplicated compounds **11**-**13**.


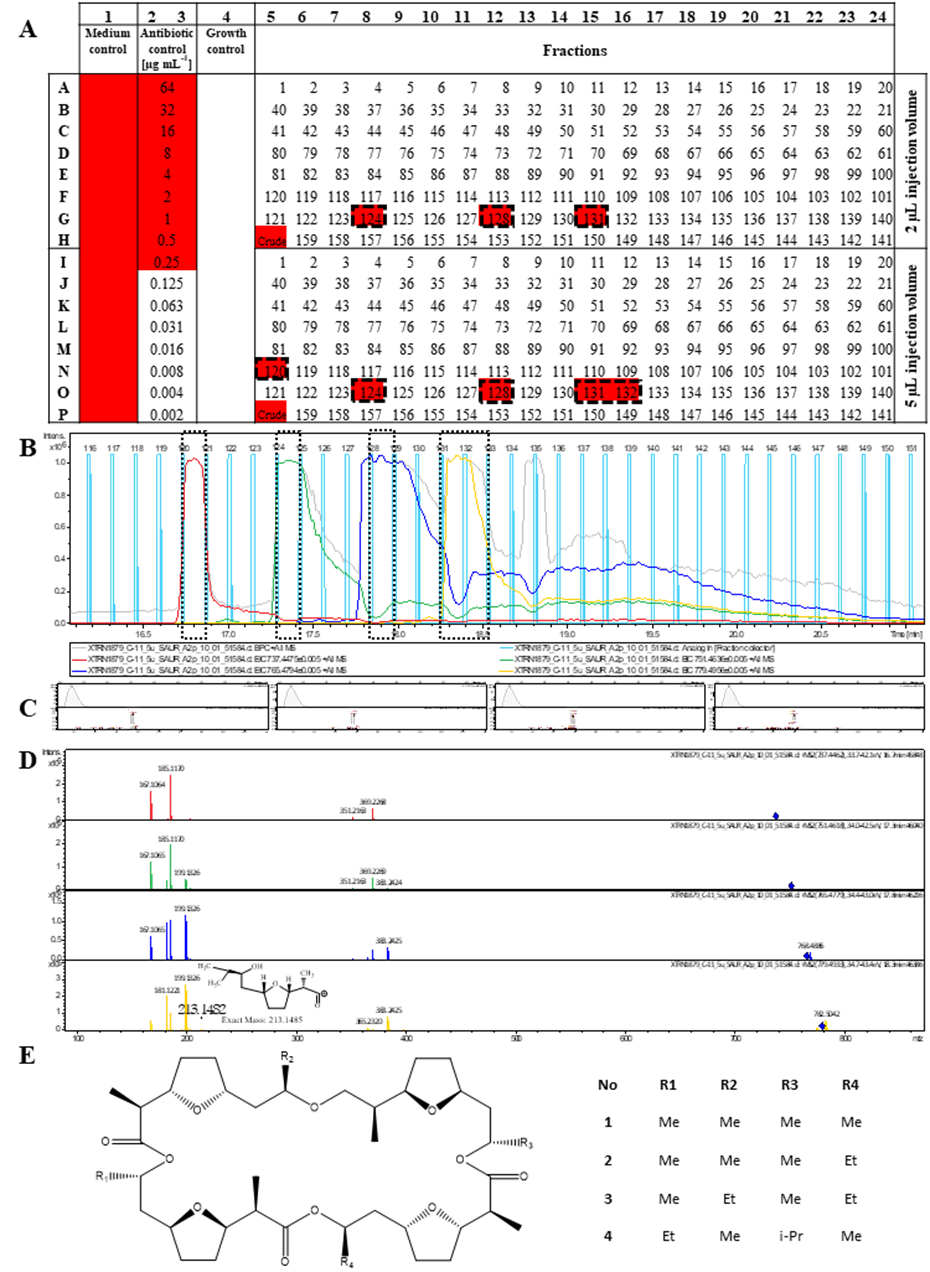


**Supplementary Fig. S9. (A)** Assay read-out of μ-fractionation plate of strain FHG110508 against *Staphylococcus aureus* ATCC 25923. Fractions are numbered and those causing at least 70% rel. growth inhibition were considered “active” and marked red. Column 1: medium control; Column 2+3: antibiotic standard (gentamycin); Column 4: growth control. Area AH05-AH24: 2 μL injection volume; Area IP05-IP24: 5 μL injection; Crude: crude extract as a control. **(B)** Overlaid Base peak Chromatograms (grey), Fraction collector analog signals (light blue bars) and extracted ion chromatogram s of *m/z* 737.4475±0.005 [M+H]^+^ (**14**, red), *m/z* 751.4636±0.005 [M+H]^+^ (**15**, green), *m/z* 765.4794±0.005 [M+H]^+^ (**16**, blue), and *m/z* 779.4956±0.005 [M+H]^+^ (**17**, yellow) of the 50 fold concentrated extract (in MeOH) with 5μL injection volume. **(C)** UV and MS spectrum of fractions 120, 124, 128 and 131-132 (from left to right). **(D)** MS/MS fragmentation of the precursor ion at *m/z* 737.4462 [M+H]^+^, *m/z* 751.4618 [M+H]^+^, *m/z* 765.4779 [M+H]^+^, and *m/z* 779.4933 [M+H]^+^ (dereplicated as nonactin, monactin, dinactin and macrotetrolide G, respectively) with manual annotation of the neutral loss and proposed structure of the fragment ion at *m/z* 213.1482 of parent ion at *m/z* 779.4933 indicating the presents of macrotetrolide G instead of trinactin. **(E)** Structures of all four dereplicated macrotetrolides. Me: methyl; Et: ethyl; iPr: isopropyl.


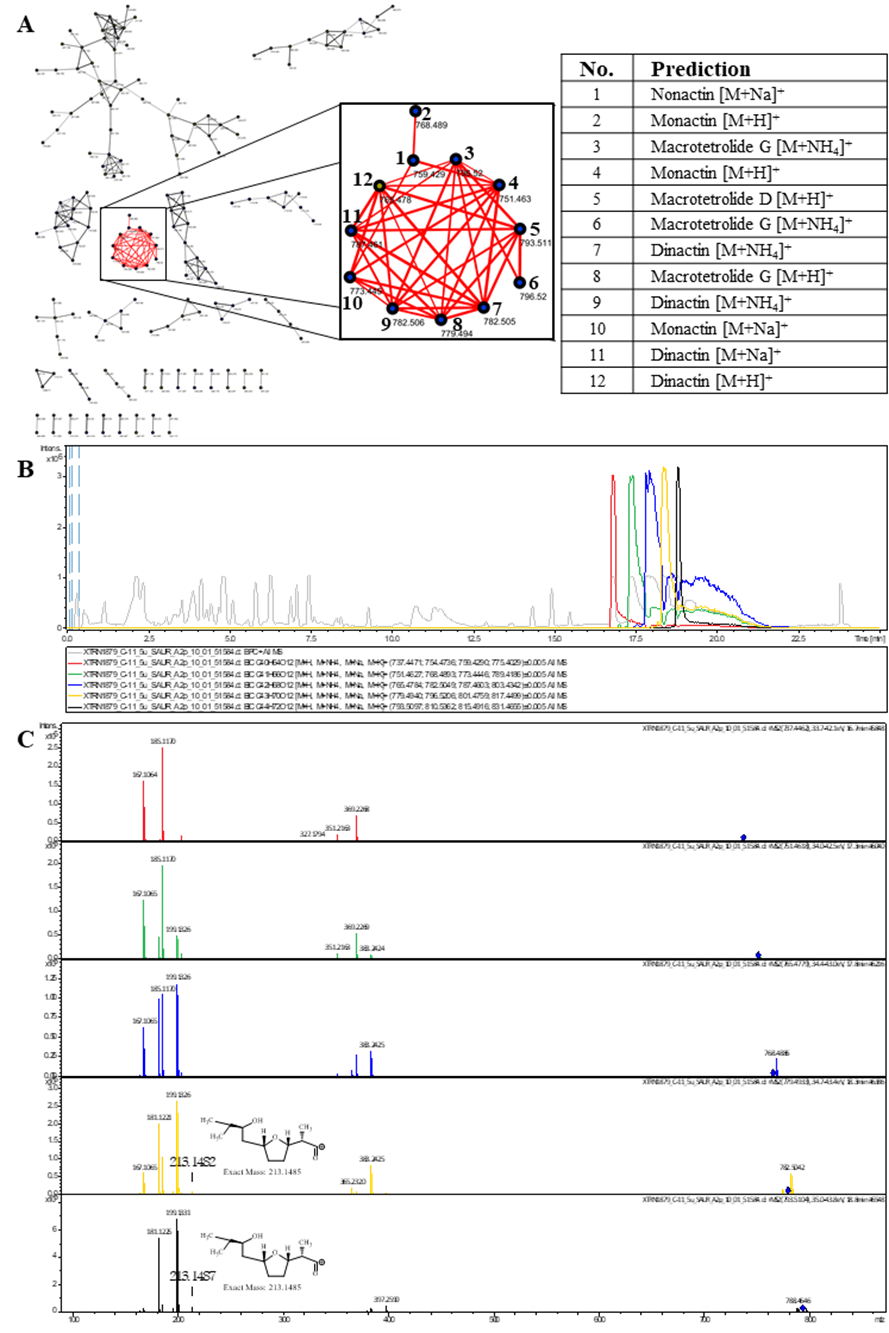


**Supplementary Fig. S10.** (**A**) MS2-network of “active” extract of FHG110508 against *Staphylococcus aureus* ATCC 25923 with focus on the cluster representing all five detected macrotetrolide derivatives and their adduct ions (dots of parent ions found as hits in our internal database or AntiBase are marked in gold). (**B**) Overlaid Base peak Chromatogram (grey) and extracted ion chromatograms of macrotetrolide **14**-**18** (**14** *m/z* 737.4462 [M+H]^+^, C_40_H_65_O_12_^+^ (red); **15** *m/z* 751.4618 [M+H]^+^, C_41_H_67_O_12_^+^ (green); **16** *m/z* 765.4779 [M+H]^+^, C_42_H_69_O_12_^+^ (blue); **17** *m/z* 779.4933 [M+H]^+^, C_43_H_71_O_12_^+^ (yellow); **18** *m/z* 793.5104 [M+H]^+^, C_44_H_73_O_12_^+^ (black)) of the 50 fold concentrated extract (in MeOH) with 5 μL injection volume. (**C**) MS/MS fragmentation of the precursor ions **14**-**18** dereplicated as nonactin, monactin, dinactin, macrotetrolide G, and macrotetrolide D, respectively, with manual annotation of the neutral loss and proposed structure of the fragment ion at *m/z* 213.1482 of parent ion at *m/z* 779.4933 indicating the presents of macrotetrolide G instead of trinactin and fragment ion at *m/z* 213.1487 of parent ion at *m/z* 793.5104 indicating the presents of macrotetrolide D instead of tetranactin.

**References**

Caballero-Aguilara, L.M., Duchib, S., Quigleyd, A., Onofrillob, C., Di Bellab, C., and Moultona, S.E. (2021) Microencapsulation of Growth Factors by microfluidic system. *MethodsX, doi:* 10.1016/j.mex.2021.101324.

Duetz, W.A., Rüedi, L., Hermann, R., O'Connor, K., Büchs, J., and Witholt, B. (2000) Methods for Intense Aeration, Growth, Storage, and Replication of Bacterial Strains in Microtiter Plates. *Applied and Environmental Microbiology, doi:* 10.1128/AEM.66.6.2641-2646.2000.

Marner, M., Patras, M.A., Kurz, M., Zubeil, F., Förster, F., Schuler, S.*, et al.* (2020) Molecular Networking-Guided Discovery and Characterization of Stechlisins, a Group of Cyclic Lipopeptides from a Pseudomonas sp. *Journal of Natural Products, doi:* 10.1021/acs.jnatprod.0c00263.

Oberpaul, M., Zumkeller, C.M., Culver, T., Spohn, M., Mihajlovic, S., Leis, B.*, et al.* (2020) High-Throughput Cultivation for the Selective Isolation of Acidobacteria From Termite Nests. *Frontiers in Microbiology, doi:* 10.3389/fmicb.2020.597628.
